# Circulating cell‐free HPV DNA is a strong marker for disease severity in cervical cancer

**DOI:** 10.1002/1878-0261.13538

**Published:** 2023-11-01

**Authors:** Sara Bønløkke, Torben Steiniche, Boe Sandahl Sorensen, Gitte‐Bettina Nyvang, Jacob Christian Lindegaard, Jan Blaakær, Jesper Bertelsen, Katrine Fuglsang, Mikael Lenz Strube, Suzan Lenz, Magnus Stougaard

**Affiliations:** ^1^ Department of Clinical Medicine Aarhus University Denmark; ^2^ Department of Pathology Aarhus University Hospital Denmark; ^3^ Department of Clinical Biochemistry Aarhus University Hospital Denmark; ^4^ Department of Oncology Odense University Hospital Denmark; ^5^ Department of Oncology Aarhus University Hospital Denmark; ^6^ Department of Obstetrics and Gynecology Odense University Hospital Denmark; ^7^ Department of Clinical Research University of Southern Denmark Odense M Denmark; ^8^ Department of Obstetrics and Gynecology Aarhus University Hospital Denmark; ^9^ DTU Bioengineering Technical University of Denmark Kongens Lyngby Denmark; ^10^ Private Gynecological Clinic “Suzan Lenz Gynækolog” Copenhagen Denmark

**Keywords:** cervical cancer, circulating HPV DNA, HPV, HPV integration, next‐generation sequencing, Sedlis criteria

## Abstract

For cervical cancer (CC), circulating cell‐free HPV DNA (ccfHPV) may establish disease severity. Furthermore, HPV integration has been correlated to viral load and survival. In this study, pre‐treatment plasma from 139 CC cases (50 primary surgery patients, 22 primary surgery + adjuvant oncological therapy patients, and 67 primary oncological therapy patients) was collected (2018–2020). Furthermore, plasma from 25 cervical intraepithelial neoplasia grade 3 patients and 15 healthy women (negative controls) were collected. Two next‐generation sequencing (NGS) panels were used to establish ccfHPV presence and human papillomavirus type 16 (HPV16) integration status. ccfHPV was detected in four primary surgery (8.0%), eight primary surgery + adjuvant oncology (36.4%), and 54 primary oncology (80.6%) patients. For primary oncology patients with HPV16‐related cancer (*n* = 37), more ccfHPV^neg^ than ccfHPV^pos^ patients had HPV16 integration (*P* = 0.04), and in patients with HPV16 integration (*n* = 13), ccfHPV^pos^ patients had higher disease stages than ccfHPV^neg^ patients (*P* = 0.05). In summary, ccfHPV presence is related to disease severity and may add to the debated Sedlis criteria used for identifying patients for adjuvant oncological therapy. However, ccfHPV detection is influenced by HPV integration status and disease stage, and these factors need to be considered in ccfHPV^neg^ patients.

## Graphical abstract

For cervical cancer (CC), circulating cell‐free HPV DNA (ccfHPV) may establish disease severity. Using targeted next‐generation sequencing of cell‐free DNA, we established ccfHPV presence and HPV16 integration status in CC patients. Our findings suggest that ccfHPV detection is related to disease severity but also influenced by HPV integration status and disease stage.

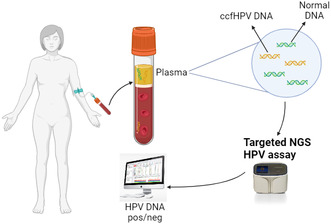

**Abbreviations**
AC: adenocarcinoma
ASC: adenosquamous carcinoma
CC: cervical cancer
ccfHPV DNA: circulating cell‐free HPV DNA
ccfHPV^neg^
: ccfHPV DNA negative
ccfHPV^pos^
: ccfHPV DNA positive
cfDNA: cell‐free DNA
CIN3: cervical intraepithelial lesion grade 3
ctDNA: circulating tumor DNA
ddPCR: digital droplet PCR
DOI: depth of invasion
FFPE: formalin‐fixed and paraffin‐embedded
FIGO: International Federation of Gynecology and Obstetrics
HPV: Human papillomavirus
LACC: locally advanced cervical cancer
LoD: limit of detection
LVSI: lymphovascular space invasion
NGS: next‐generation sequencing
NPV: negative predictive value
PPV: positive predictive value
SCC: squamous cell carcinoma

## 1 Introduction

Human papillomavirus (HPV) causes 99.7% of all cervical cancers (CC) [1]. For CC, the disease stage [2] is the most important predictor of survival and is therefore used to guide treatment, where patients with localized early‐stage disease are treated with primary surgery and patients with locally advanced cervical cancer (LACC) are treated with primary chemoradiation and brachytherapy. Further, for patients initially surgically treated, post‐operative histopathological findings are used to guide potential adjuvant oncological therapy decision [3]. These findings are categorized as either intermediate‐risk factors according to Sedlis criteria, which includes information on tumoral depth of invasion (DOI), lymphovascular space invasion (LVSI), and tumor size [4] or high‐risk factors according to Peter's criteria, which includes lymph node metastasis, positive surgical margins, and parametrial involvement [5]. While the impact of high‐risk factors on disease recurrence is indisputable [5, 6], controversies remain regarding adjuvant treatment after surgery for patients with localized CC who meet the Sedlis criteria [7, 8, 9]. Since identifying patients who will benefit from adjuvant treatment is critical to reduce morbidity and mortality from CC, improvements in the criteria used to guide adjuvant treatment decision are needed.

In recent years, cell‐free DNA (cfDNA), comprising degraded DNA fragments released to the circulation by cell death and active secretion processes [10, 11], has been explored as a biomarker for detecting and monitoring cancer [12, 13, 14]. cfDNA released from tumor cells is known as circulating tumor DNA (ctDNA), and this is already widely used for monitoring disease in various cancer types [13, 15, 16]. Recent research suggests the possible clinical value of circulating cell‐free HPV DNA (ccfHPV DNA) in patients with HPV‐related cancers [17, 18, 19, 20, 21, 22, 23, 24, 25], and we therefore conducted a proof‐of‐concept study to investigate the diagnostic value of ccfHPV16 and 18 DNA in CC patients [26]. In agreement with previous findings, we found that ccfHPV DNA detection and quantity are closely related to different clinical parameters such as disease stage and tumor size and may thus be a promising tool to establish tumor burden in patients with LACC [17, 23, 25, 27]. However, for patients with lower disease burden, ccfHPV DNA positivity is significantly lower [18, 24, 25], and for women with only precancerous cervical lesions, the biomarker is non‐detectable [24, 25], demonstrating that a certain disease burden is required for HPV DNA to be detected or even present in the blood circulation.

In two previous studies published by our group, we validated a targeted next‐generation sequencing (NGS) panel for both HPV detection and genotyping, as well as evaluation of HPV integration status [28, 29], which has been shown to reflect viral load [30, 31] and prognosis [30, 31, 32, 33] in CC. Integration of HPV DNA was previously considered a critical event in the process of cervical carcinogenesis [34, 35, 36]. However, studies have detected non‐integrated HPV DNA in a significant number of CCs, either as an episome or as a combination of both integrated and episomal forms (mixed stage) [30, 37, 38, 39], and studies have shown a great variation in the frequency of HPV integration according to HPV genotype [30, 40, 41], presumably because of phylogenetic differences between genotypes. Thus, consistent integration of HPV18 [30, 40, 41] and HPV45 [30, 41] has been observed in almost all CCs, whereas, for example, HPV31, HPV33 [30, 41], and HPV16 integration [30, 40, 41] is less frequent. In the present study, we aimed at using our NGS panel [29] (hereafter referred to as “the NGS HPV genotyping panel”) as well as a recently developed comprehensive HPV16 NGS panel for assessment of integration status on cervical tissue and plasma samples from 139 women with CC. With our analyses, we sought to (I) test the performance of our NGS HPV genotyping panel for ccfHPV DNA detection, (II) test the performance of our NGS HPV16 panel for assessing HPV16 integration status, and (III) assess the value of ccfHPV DNA as a marker for disease severity. To our knowledge, this is the first study to combine findings on ccfHPV DNA in plasma with the integration status from patients with HPV‐related CC.

## 2 Materials and methods

### 2.1 Participants and tissue/blood sample collection

This study is the further development of our previous proof‐of‐concept study on ccfHPV DNA in plasma from CC patients [26]. Thus, the inclusion of the total study cohort included in the present study is described in detail in Bønløkke et al. [26]. In short, a total of 262 patients diagnosed with CC between June 2018 and October 2020 at Aarhus University Hospital or between January 2019 and January 2020 at Odense University Hospital were invited to participate, and after two rounds of exclusion, the total case cohort comprised 141 CC patients with stage IA1‐IVB disease (according to the International Federation of Gynecology and Obstetrics, FIGO 2018 [2]) (fig. 1 in Bønløkke et al. [26]). However, after this study's analyses of plasma samples from all 141 patients, no human or ccfHPV DNA was detected in plasma from two patients, leaving 139 CC patients in the final case cohort. We subdivided the cohort into three groups: patients with localized disease treated with primary surgery (i.e., primary surgery subgroup, *n* = 50), patients with localized disease treated with primary surgery and adjuvant oncological therapy (surgery followed by adjuvant chemoradiation or surgery followed by adjuvant radiotherapy) (i.e., primary surgery + adjuvant oncology subgroup, *n* = 22), and LACC patients treated with primary oncological therapy (chemoradiation, neoadjuvant chemotherapy followed by radiotherapy, or neoadjuvant chemotherapy followed by chemoradiation) (i.e., primary oncology subgroup, *n* = 67). Table S1 provides clinical and biological data on the study cohort.

We collected blood samples from all cases at the time of diagnosis and prior to treatment initiation (i.e., surgery or oncological therapy). Furthermore, 25 women diagnosed with cervical intraepithelial lesion grade 3 (CIN3) and a negative control group represented by 15 healthy anonymous women (negative controls) were included. CIN3 patients were included at Regional Hospital Randers (see fig. S1 in [26]). The CIN3 patients were included to examine whether ccfHPV DNA is detectable in precancerous lesions and thus may be useful for screening. Negative controls were included in connection with blood donation at the Department of Clinical Immunology at Aarhus University Hospital. These women all had a negative cervical smear within the past 3 years.

For CC patients and CIN3 patients, formalin‐fixed and paraffin‐embedded (FFPE) cervical tissue samples were collected for HPV analysis.

### 2.2 DNA extraction and HPV analyses

#### 2.2.1 DNA extraction from tissue

We performed HPV genotyping on cervical tissue from all 139 CC patients and all 25 CIN3 patients. For the protocol on the extraction of DNA from cervical tissue, see Bønløkke et al. [26]. Briefly, for each patient, the FFPE cervical tissue sample was used to cut and collect three to four 10 μm thick sections in a sterile tube. DNA extraction was performed using the QIAsymphony DSP DNA Mini Kit, version 1 (Qiagen, Venlo, The Netherlands).

#### 2.2.2 Cell‐free DNA extraction from plasma

Preparation of blood samples and extraction of plasma and cfDNA is described in detail in Bønløkke et al. [26]. Briefly, a total of 30 mL full blood was collected from each participant in Cell‐Free DNA BCT® collection tubes (Streck, La Vista, NE, USA), and after DNA extraction, cfDNA was extracted from 5 mL of plasma using QIAamp Circulating Nucleic Acid Kit (QIAGEN, Hilden, Germany) following the manufacturer's instructions.

#### 2.2.3 INNO‐LiPA HPV genotyping extra II (INNO‐LiPA) for tissue analyses

Human papillomavirus DNA in all tissue samples was detected by two different HPV genotyping assays: the INNO‐LiPA assay (Fujirebio Europe, Ghent, Belgium), and the NGS HPV genotyping panel. We performed the analyses on the INNO‐LiPA according to the manufacturer's instructions. The INNO‐LiPA uses the SPF10 primer set to amplify a 65 base pair (bp) region in the L1 gene, followed by HPV genotyping by reverse hybridization. The assay can detect 32 HPV genotypes, including the high‐risk HPV genotypes (16, 18, 31, 33, 35, 39, 45, 51, 52, 56, 58, 59), most of the potentially high‐risk HPV genotypes (25, 53, 66, 68, 70, 73, 82), and the low‐risk HPV genotypes (6, 11, 40, 42, 43, 44, 54, 61, 62, 67, 81, 83, 89).

#### 2.2.4 NGS HPV genotyping panel design, sample preparation, and sequencing for tissue and plasma analyses

The designing of the NGS HPV genotyping panel is described in detail in Andersen et al. [29]. This panel includes the 25 IARC‐classified carcinogenic (HPV16, 18, 31, 33, 35, 39, 45, 51, 52, 56, 58, 59), probably carcinogenic (HPV68), and possibly carcinogenic (HPV26, 30, 34, 53, 66, 67, 69, 70, 73, 82, 85, 97) HPV genotypes and the two most common low‐risk HPV genotypes HPV6 and HPV11 [42]. For each of these 27 HPV genotypes, eight areas were detected; two areas in each of the E6 and E7 oncogenes, one area in the L1 capsid gene, and three areas in the E2 gene. Furthermore, as a quality assessment and to confirm the presence of human DNA in the tested samples, the panel includes five human reference genes (BTF3, PABPN1, PPIE, RAB1B, and SRSF3). The panel was designed with short amplicons of approximately 90 base pairs using the circulating free DNA pipeline of the Ampliseq Designer. The samples were prepared for sequencing using the Ion AmpliSeq Library Kit 2.0 (Thermo Fisher Scientific, Roskilde, Denmark) according to the manufacturer's protocol and with 24 PCR cycles. Input DNA was 60 ng for tissue and in the range of 2.6–60 ng for plasma. Samples were labeled with Ion Xpress Barcode Adapters (Thermo Fisher Scientific, Roskilde, Denmark). Before pooling the libraries for template preparation and sequencing, they were quantified using the Ion Library TaqMan Quantitation Kit (Thermo Fisher Scientific, Roskilde, Denmark) and diluted to 40 pm. Template preparation was performed with the Ion 510 & Ion 520 & Ion 530 Kit—Chef (Thermo Fisher Scientific, Roskilde, Denmark) by using the Ion Chef System (Thermo Fisher Scientific, Roskilde, Denmark). The samples were sequenced using the Ion GeneStudio S5 System (Thermo Fisher Scientific, Roskilde, Denmark), and the coverage analysis plugin used for the data analysis was set to a minimum amplicon length of 50 base pairs.

#### 2.2.5 NGS HPV genotyping data analysis on tissue and plasma

Except for the calculation of the limit of detection (LoD) for HPV positivity, NGS data analyses were done essentially as described in Andersen et al. [29]. Because of the different background noise, the LoD was calculated separately for tissue and plasma using tissue from non‐HPV16‐positive patients and plasma from negative controls, respectively. For a thorough description of the approaches used to determine LoD for tissue, see Table S2 and the corresponding footnotes. Thus, LoD for tissue was 20.4 reads, and therefore, cut‐off for HPV DNA positivity was established as ≥ 2 amplicons for the same HPV genotype with > 20 reads. However, none of the results for HPV DNA for cervical tissue were at all near this cut‐off (data not shown), making this cut‐off mostly theoretical.

For plasma, we used a similar approach, and for a thorough description of the approaches used to determine LoD, see Table S3 and the corresponding footnotes. Thus, LoD for plasma was 4 reads, and therefore, cut‐off for HPV DNA positivity was established as ≥ 2 amplicons for the same HPV genotype with > 4 reads. However, data from plasma analyses of the 14 ccfHPV DNA‐positive cases and the 16 ccfHPV DNA‐negative cases with results closest to this cut‐off value (Tables S4 and S5) show that none of the results for ccfHPV DNA were near this cut‐off, also making this cut‐off value mostly theoretical.

#### 2.2.6 NGS HPV16 panel design and sequencing on HPV16‐positive tissue

Besides analyzing cervical tissue samples with INNO‐LiPA and the NGS HPV genotyping panel, the tissue samples from the 67 CC patients positive for HPV16 in their tissue sample (only single genotype infections) were analyzed with our recently developed NGS HPV16 panel. As previously described, studies have shown that integration of HPV DNA into the human genome is a negative prognostic sign [30, 31, 32, 33], and thus, this panel enabled a thorough evaluation of HPV16 integration status for a tested sample. Upon integration of the virus, disruption and thus partial or complete loss of specific HPV genes has been shown to frequently occur [30, 43, 44]. The breakpoints occur within the early E1–E5 or late L1–L2 genes, most frequently in the E1/E2 genes, which causes constitutive expression of the E6/E7 oncoproteins [30].

Thus, for CC, HPV DNA can be either completely integrated or non‐integrated (episomal or mixed stage) [30, 37, 38, 39], and therefore, the NGS HPV16 panel were designed with 104 amplicons covering 97% of the HPV16 genome. As with the NGS HPV genotyping panel, the NGS HPV16 panel was designed using the AmpliSeq Designer (Thermo Fisher Scientific, Roskilde, Denmark), and to confirm the presence of human DNA in each sample, the panel included the same human reference genes as the NGS HPV genotyping panel (BTF3, PABPN1, PPIE, RAB1B, and SRSF3). The panel was also designed with short amplicons of approximately 90 base pairs using the circulating free DNA pipeline of the Ampliseq Designer. Sample preparation was performed as for the NGS HPV genotyping panel analyses.

#### 2.2.7 NGS HPV16 panel data analysis on HPV16‐positive tissue

Data analyses were done as described in Section 2.2.5. for the NGS HPV genotyping panel. As with the NGS genotyping panel, a similar approach was used to calculate LoD for HPV positivity in tissue for the NGS HPV16 panel, and for a thorough description of the approaches used to determine LoD, see Table S6 and the corresponding footnotes. Thus, cut‐off for HPV16 DNA positivity was established as ≥ 2 amplicons with > 15 reads. However, as for the cut‐offs for ccfHPV DNA and HPV DNA positivity with the NGS HPV genotyping panel, none of the results for HPV16 DNA were at all near this cut‐off (data not shown), also making this cut‐off mostly theoretical.

Analyses using this panel were performed with the purpose of assessing HPV16 integration status. Therefore, based on the assumption that preferential deletion of part of a gene in the HPV genome (most often reported in E1, E2, L1, or L2 [30]) will cause complete absence of the gene sequence concerned and thus complete integration, complete integration was defined as ≥ 2 consecutive amplicons with ≤ 15 reads, and non‐integration was defined as < 2 consecutive amplicons with < 15 reads.

### 2.3 Statistics

Detection rate of ccfHPV DNA was defined as the number of ccfHPV DNA‐positive (ccfHPV^pos^) plasma samples from CC patients divided by the total number of CC patients. By means of ccfHPV DNA results on patients from the primary surgery subgroup and the primary oncology subgroup (*n* = 117), sensitivity, specificity, positive predictive value (PPV), and negative predictive value (NPV) for identifying patients requiring primary oncological therapy with the NGS HPV genotyping panel were calculated based on the assumption that true positives are ccfHPV^pos^ primary oncology patients, true negatives are ccfHPV DNA‐negative (ccfHPV^neg^) primary surgery patients, false positives are ccfHPV^pos^ primary surgery patients, and false negatives are ccfHPV^neg^ primary oncology patients. Fisher's exact test was used to compare categorical variables between patients, and Student's *t*‐test was used to compare tumor size between patients. We considered a *P*‐value of ≤ 0.05 as statistically significant.

### 2.4 Ethics

The study methodologies conformed to the standards set by the Declaration of Helsinki and were approved by the Central Denmark Region Committees on Health Research Ethics (journal number: 1‐10‐72‐381‐17) and the Danish Data Protection Agency (journal number: 1‐16‐02‐782‐17). The experiments were undertaken with the understanding and written consent of each subject. Furthermore, all subjects involved in the study have given their consent for publication of data and results.

## 3 Results

### 3.1 Patient characteristics and concordance in HPV genotyping on cervical tissue

The case cohort comprised 139 HPV‐positive CC patients, 50 patients (36.0%) in the primary surgery subgroup, 22 patients (15.8%) in the primary surgery + adjuvant oncology subgroup, and 67 patients (48.2%) in the primary oncology subgroup (Table S1). Furthermore, 25 CIN3 patients and a control cohort comprising 15 healthy women were included (fig. S1 in Bønløkke et al. [26]). Histologically, the CC cases comprised 106 (76.3%) squamous cell carcinomas (SCC), 26 (18.7%) adenocarcinomas (AC), and seven (5.0%) adenosquamous carcinomas (ASC) (Table S1).

As described previously, HPV genotyping analyses on cervical tissue from cases were performed with both the INNO‐LiPA assay and the NGS HPV genotyping panel. When comparing HPV genotyping results, we only compared results on genotypes included in both assays, and thus, 137 samples were comparable. Of these, there was a complete agreement between the two assays in 130 (94.9%) samples and partial agreement in the remaining seven (5.1%) samples, meaning that one of the assays detected one genotype and the other assay detected the same genotype as well as one other genotype (Table S1). For the two non‐comparable samples (pt. no 76 and no 102, respectively), the INNO‐LiPA detected HPV16 and HPV positive (type unspecified), respectively, whereas the NGS assay detected HPV16 + HPV67 and HPV30, respectively (Table S1). Thus, since the INNO‐LiPA assay cannot genotype HPV67 and HPV30, these results were most likely also in complete agreement. For the further analyses in this study, HPV genotyping results refer to the results of the NGS HPV genotyping panel. Table 1 shows patient and tumor characteristics for the three subgroups, and furthermore, all relevant clinical and biological data are given in Table S1.

**Table 1:** Patient and tumor characteristics for the three subgroups (*N* = 139).

Patients	Primary surgery subgroup, *N* = 50	Primary surgery + adjuvant oncology subgroup, *N* = 22	Primary oncology subgroup, *N* = 67
Age (mean ± SD)	45.7 ± 12.0	48.6 ± 13.3	54.8 ± 15.7
Histology	***n* (%)**	***n* (%)**	***n* (%)**
SCC	29 (58)	15 (68.2)	62 (92.5)
AC	16 (32.0)	5 (22.7)	5 (7.5)
ASC	5 (10.0)	2 (9.1)	0 (0.0)
FIGO 2018^a^	***n* (%)**	***n* (%)**	***n* (%)**
IA1	1 (2.0)	0 (0.0)	0 (0.0)
IA2	15 (30.0)	0 (0.0)	0 (0.0)
IB1	16 (32.0)	0 (0.0)	0 (0.0)
IB2	16 (32.0)	9 (40.9)	0 (0.0)
IB3	2 (4.0)	1 (4.5)	3 (4.5)
IIA1	0 (0.0)	2 (9.1)	0 (0.0)
IIB	0 (0.0)	0 (0.0)	26 (38.8)
IIIB	0 (0.0)	0 (0.0)	1 (1.5)
IIIC1	0 (0.0)	0 (40.9)	32 (47.8)
IIIC2	0 (0.0)	1 (4.5)	3 (4.5)
IVA	0 (0.0)	0 (0.0)	2 (3.0)
*T*‐score (mean ± SD)^b^	1.1 ± 0.7	2.2 ± 0.4	5.9 ± 3.1
Tumor size (mm) (mean ± SD)^c^	17.2 ± 9.1	27.4 ± 7.6	52.3 ± 20.8
HPV genotype tissue^d^	***n* (%)**	***n* (%)**	***n* (%)**
16	23 (46.0)	7 (31.8)	37 (55.2)
18	11 (22.0)	5 (22.7)	6 (9.0)
31	2 (4.0)	1 (4.5)	3 (4.5)
33	1 (2.0)	2 (9.1)	4 (6.0)
45	5 (10.0)	2 (9.1)	7 (10.4)
Other	3 (6.0)	3 (13.6)	5 (7.5)
Multiple	5 (10.0)	2 (9.1)	5 (7.5)

a Disease stage according to FIGO 2018 [2]. For patients having been re‐staged after surgery, the re‐staged stage is the one listed. For primary stage, see Table S1.

b *T*‐score according to Lindegaard et al. [55]. The scoring system is developed for CC patients with stage IB‐IVB disease, giving patients with stage IB1, IB2, and IB3 a *T*‐score of 1, 2, and 3 respectively. Thus, for patients with stage IA1 and IA2 disease, we made a presumption that stage IA1 equals a *T*‐score of 0.25 and stage IA2 equals a *T*‐score of 0.5. For early‐stage patients having been re‐staged after surgery, the re‐staged stage is the one used to determine *T*‐score. For primary disease stage, see Table S1.

c Large diameter of tumor. For the primary surgery subgroup and the primary surgery + adjuvant oncology subgroup, tumor size was evaluated pathologically *after* surgery based on the removed tissue. For the primary oncology subgroup, tumor size was evaluated based on clinical examination or magnetic resonance imaging (MRI) prior to treatment.

d Cervical tissue samples tested with the HPV NGS genotyping panel.

### 3.2 Detection of ccfHPV DNA using NGS HPV genotyping panel

In our previous study [26], digital droplet PCR (ddPCR) was used for a qualitative and quantitative assessment of ccfHPV DNA in plasma from 60 HPV16‐ or HPV18‐positive CC patients, eight CIN3 patients, and 15 negative controls. Here, we found that 19 LACC patients treated with primary oncological therapy (63.3%) and three early‐stage patients treated with primary surgery (10.0%) were positive for ccfHPV16‐ or 18 DNA, and both CIN3 patients and negative controls were negative for ccfHPV DNA. To see if the NGS HPV genotyping panel could perform similarly to the ddPCR assay, the current study used the NGS genotyping panel to analyze cfDNA from the same 60 patients and the remaining 79 patients in the case cohort. For the 60 patients also included in Bønløkke et al. [26], we found that 25 LACC patients (83.3%) and two early‐stage patients (6.7%) were positive for ccfHPV DNA with the NGS panel (Table S7). Thus, the detection rate for LACC patients was increased with the NGS panel, and therefore, our following ccfHPV DNA analyses on the remaining patients were performed with the NGS panel. For our total study cohort of CC patients, ccfHPV DNA was detected in 66 patients (47.5%): four patients (8.0%) in the primary surgery subgroup; eight in the primary surgery + adjuvant oncology subgroup (36.4%), four (33.3%) intermediate‐risk patients (*P* = 0.04) and four (40.0%) high‐risk patients (*P =* 0.02); and 54 (80.6%) in the primary oncology subgroup (*P =* 0.00) (Table 2 and Table S7).

**Table 2:** Qualitative detection of ccfHPV DNA according to treatment (*N* = 139).

	ccfHPV DNA^pos^, *n* (%)	ccfHPVDNA^neg^, *n* (%)	*P‐*value
Primary surgery subgroup^a^ (*N* = 50)	4 (8.0)	46 (92.0)	
Primary surgery + adjuvant oncology subgroup (*N* = 22)			
Intermediate‐risk patients (*N* = 12)	4 (33.3)	8 (66.7)	0.04^b^
High‐risk patients (*N* = 10)	4 (40.0)	6 (60.0)	0.02^c^
Primary oncology subgroup^a^			
LACC patients (*N* = 67)	54 (80.6)	13 (19.4)	0.00^d^

a Only patients from the primary surgery subgroup and patients from the primary oncology subgroup (*n* = 117) were used to measure sensitivity, specificity, PPV, and NPV for using ccfHPV DNA as a marker for identifying patients requiring primary oncological therapy.

b Fisher's exact test comparing primary surgery subgroup vs. intermediate‐risk patients in the primary surgery + adjuvant oncology subgroup.

c Fisher's exact test comparing primary surgery subgroup vs. high‐risk patients in the primary surgery + adjuvant oncology subgroup.

d Fisher's exact test comparing primary surgery subgroup vs. primary oncology subgroup.

Because of the previously described controversies regarding adjuvant treatment after surgery for patients with localized CC who meet the Sedlis criteria, that is, intermediate‐risk patients [7, 8], and since there is not a clear consensus on whether oncological therapy is sufficient treatment for high‐risk patients, only patients from the primary surgery subgroup and patients from the primary oncology subgroup (*n* = 117) were used to measure sensitivity, specificity, PPV, and NPV of the NGS genotyping panel for identifying patients requiring primary oncological therapy. Based on the previously mentioned assumptions on true and false positives and the true and false negatives, our NGS HPV genotyping panel showed a sensitivity of 80.6%, a specificity of 92.0%, a PPV of 93.1%, and a NPV of 78.9% (Table 2). However, it is worth mentioning that for the four ccfHPV^pos^ patients in the primary surgery subgroup, two of these patients (50.0%) developed a disease recurrence (pt no 22 and no 71) within 19 and 6 months after the diagnosis, respectively, whereas this was only the case for one (2.2%) of the 46 ccfHPV^neg^ primary surgery patients (pt no 3 within 25 months after the diagnosis). Analyses of cfDNA from controls and CIN3 patients were all negative for ccfHPV DNA (Table S3).

### 3.3 ccfHPV DNA results according to treatment and clinical parameters

#### 3.3.1 ccfHPV DNA detection according to treatment

Since FIGO stage and consequently treatment modality is the most important predictor of survival from CC, and since the validity of the Sedlis criteria used to guide adjuvant treatment after surgery for patients with intermediate‐risk factors is much debated, we examined whether ccfHPV DNA detection rate varies between the intermediate‐risk, high‐risk, and LACC patients, respectively. Table 2 shows the qualitative detection of ccfHPV DNA for these patients according to treatment. Interestingly, for intermediate‐ and high‐risk patients, respectively, four (33.3%) and four (40.0%) were ccfHPV^pos^ at diagnosis. For LACC patients, 54 (80.6%) were ccfHPV^pos^ at diagnosis.

#### 3.3.2 ccfHPV DNA status according to clinical parameters

Table 3 shows the clinical parameters based on ccfHPV DNA status for all three subgroups. To examine possible explanations for the 13 ccfHPV^neg^ primary oncology patients, which we regard as false negatives, we used these patients as our reference group to examine whether they differed significantly from ccfHPV^pos^ primary oncology patients on one or more clinical parameters (Table 3). As previously described, HPV18 and HPV45 show phylogenetic similarities and are almost always completely integrated, whereas the remaining HPV genotypes can be both completely integrated and non‐integrated [30, 40, 41]. Thus, to examine the possible influence of HPV integration, we merged HPV18 and HPV45 when comparing HPV genotyping results. Overall, when comparing both histology (SCC vs. AC/ASC), HPV genotype (HPV18/45 vs. all other HPV genotyping results), tumor size, and FIGO stage, no significant differences between ccfHPV^neg^ and ccfHPV^pos^ primary oncology patients were observed (*P* = 1.00, *P* = 0.26, *P* = 0.61, and *P* = 0.64, respectively).

**Table 3:** Clinical parameters based on ccfHPV DNA status.

	ccfHPV^pos^ primary surgery^a^ (*n* = 4)	ccfHPV^neg^ primary surgery^a^ (*n* = 46)	ccfHPV^pos^ primary surgery + adj^b^ (*n* = 8)	ccfHPV^neg^ primary surgery + adj^b^ (*n* = 14)	ccfHPV^pos^ primary oncology^c^ (*n* = 54)	ccfHPV^neg^ primary oncology^c^ (*n* = 13)	*P*‐value^d^
Histology	***n* (%)**	***n* (%)**	***n* (%)**	***n* (%)**	***n* (%)**	***n* (%)**	1.00^e^
SCC	3 (75.0)	26 (56.5)	7 (87.5)	8 (57.1)	50 (92.6)	12 (92.3)	
AC	1 (25.0)	15 (32.6)	0 (0.0)	5 (35.7)	4 (7.4)	1 (7.7)	
ASC	0 (0.0)	5 (10.9)	1 (12.5)	1 (7.1)	0 (0.0)	0 (0.0)	
HPV genotype^f^	***n* (%)**	***n* (%)**	***n* (%)**	***n* (%)**	***n* (%)**	***n* (%)**	0.26^g^
HPV16	3 (75.0)	20 (43.5)	4 (50.0)	3 (21.4)	32 (59.3)	5 (38.5)	
HPV18	0 (0.0)	11 (23.9)	0 (0.0)	5 (35.7)	5 (9.3)	1 (7.7)	
HPV31	0 (0.0)	2 (4.3)	0 (0.0)	1 (7.1)	3 (5.6)	1 (7.7)	
HPV33	0 (0.0)	1 (2.2)	1 (12.5)	1 (7.1)	3 (5.6)	1 (7.7)	
HPV45	1 (25.0)	4 (8.7)	1 (12.5)	1 (7.1)	4 (9.3)	3 (23.1)	
Other	0 (0.0)	3 (6.5)	1 (12.5)	2 (14.3)	4 (9.3)	0 (0.0)	
Multiple	0 (0.0)	5 (10.9)	1 (12.5)	1 (7.1)	3 (5.6)	2 (15.4)	
Tumor size (mm)	***n* (%)**	***n* (%)**	***n* (%)**	***n* (%)**	***n* (%)**	***n* (%)**	0.61^h^
< 20	1 (25.0)	32 (69.6)	0 (0.0)	0 (0.0)	0 (0.0)	0 (0.0)	
≥ 20 < 30	2 (50.0)	11 (23.9)	6 (75.0)	9 (64.3)	2 (3.7)	0 (0.0)	
≥ 30 < 40	0 (0.0)	2 (4.3)	2 (25.0)	3 (21.4)	8 (14.8)	4 (30.7)	
≥ 40	1 (25.0)	1 (2.2)	0 (0.0)	2 (14.3)	4 (7.4)	9 (69.2)	
Mean ± SD	24.75 ± 11.3	17.3 ± 8.6	22.9 ± 6.6	28.6 ± 7.9	53.6 ± 19.2	49.9 ± 22.5	
Median (IQR)	22 (7.75)	14.5 (11.5)	22 (5.25)	25 (10.5)	50.0 (22.3)	40.0 (24.0)	
FIGO 2018^i^	***n* (%)**	***n* (%)**	***n* (%)**	***n* (%)**	***n* (%)**	***n* (%)**	0.64^j^
IA1	0 (0.0)	1 (2.2)	0 (0.0)	0 (0.0)	0 (0.0)	0 (0.0)	
IA2	0 (0.0)	15 (32.6)	0 (0.0)	0 (0.0)	0 (0.0)	0 (0.0)	
IB1	1 (25.0)	15 (32.6)	0 (0.0)	0 (0.0)	0 (0.0)	0 (0.0)	
IB2	2 (50.0)	14 (30.4)	3 (37.5)	6 (42.9)	0 (0.0)	0 (0.0)	
IB3	1 (25.0)	1 (2.2)	0 (0.0)	1 (7.1)	2 (3.7)	1 (7.7)	
IIA1	0 (0.0)	0 (0.0)	1 (12.5)	1 (7.1)	0 (0.0)	0 (0.0)	
IIB	0 (0.0)	0 (0.0)	0 (0.0)	0 (0.0)	21 (38.9)	5 (38.5)	
IIIB	0 (0.0)	0 (0.0)	0 (0.0)	0 (0.0)	1 (18.5)	0 (0.0)	
IIIC1	0 (0.0)	0 (0.0)	3 (37.5)	6 (42.9)	26 (48.1)	6 (46.2)	
IIIC2	0 (0.0)	0 (0.0)	1 (12.5)	0 (0.0)	3 (5.6)	0 (0.0)	
IVA	0 (0.0)	0 (0.0)	0 (0.0)	0 (0.0)	1 (1.9)	1 (7.7)	

a ccfHPV^pos/neg^ patients referred for primary surgery, that is, primary surgery subgroup.

b ccfHPV^pos/neg^ patients referred for primary surgery and adjuvant oncological therapy, that is, primary surgery + adjuvant oncology subgroup.

c ccfHPV^pos/neg^ referred for primary oncological therapy, that is, primary oncology subgroup.

d Statistical analyses were performed for the ccfHPV^pos^ primary oncology subgroup vs. the ccfHPV^neg^ primary oncology subgroup.

e Fisher's exact test comparing SCCs vs. AC/ASCs.

f HPV genotype according to analyses with the NGS HPV genotyping panel.

g Fisher's exact test comparing HPV18/45 vs. all other HPV genotyping results.

h Two‐sample *t*‐test with unequal variance.

i Disease stage according to FIGO 2018 [2]. For patients having been re‐staged after surgery, the re‐staged stage is the one listed.

j Fisher's exact test.

### 3.4 HPV16 integration status in tissue using NGS HPV16 panel

To examine the possible impact of HPV integration more closely, DNA from tissue samples from the 67 CC cases with HPV16‐related disease (only single genotype infections) were analyzed with our recently developed and comprehensive NGS HPV16 panel (Table S8). From the 67 cases, 23 were primary surgery patients, seven were primary surgery + adjuvant oncology patients, and 37 were primary oncology patients. We found that 19 (28.4%) had complete integration, four primary surgery patients (17.4%), two primary surgery + adjuvant oncology patients (28.6%), and 13 primary oncology patients (35.1%). For integrated cases, the genes involved were E2, L2, and especially E1 (Table S8). Since previous studies have found that HPV integration causes a lower DNA viral load [30, 31], we correlated our findings on ccfHPV DNA from the primary oncology patients with HPV16‐related cancer with their HPV16 integration status to see if some of the ccfHPV^neg^ may be explained by integration and therefore low viral load causing non‐detectable ccfHPV DNA. Interestingly, we found that significantly more ccfHPV^neg^ patients than ccfHPV^pos^ patients had complete integration (*P* = 0.04) (Table 4).

**Table 4:** Correlation between ccfHPV16 detection and HPV16 integration status for ccfHPV^neg^ and ccfHPV^pos^ primary oncology patients (*N* = 37).

HPV16 integration status^a^	ccfHPV^neg^ primary oncology (*N* = 5)^b^, *n* (%)	ccfHPV^pos^ primary oncology (*N =* 32)^c^, *n* (%)	
Complete integration	4 (80.0)	9 (28.1)	*P* = 0.04^d^
Non‐integration	1 (20.0)	23 (71.9)	

a HPV16 integration status defined as follows; complete integration: ≥ 2 consecutive amplicons with 0 reads; non‐integration: < 2 consecutive amplicons with 0 reads.

b ccfHPV^neg^ primary oncology patients with HPV16‐related cancer.

c ccfHPV^pos^ primary oncology patients with HPV16‐related cancer.

d Fisher's exact test.

When assessing possible differences in disease severity in primary oncology patients with HPV16 integration, we found that ccfHPV^pos^‐integrated patients had significantly higher disease stages than ccfHPV^neg^‐integrated patients (*P* = 0.05) (Table 5), whereas no significant difference was detected for tumor size (*P* = 0.86). These data may suggest that detection of ccfHPV DNA in patients with integrated HPV is influenced by the disease stage. When comparing tumor size and FIGO stage between primary oncology patients with and without complete integration regardless of ccfHPV DNA status, no differences were seen (*P* = 0.31 and *P* = 0.97, respectively) (Tables S8 and S9), showing that integration status alone does not affect clinical parameters.

**Table 5:** Disease severity in primary oncology patients with HPV16 integration according to ccfHPV DNA detection (*n* = 13).

	ccfHPV^pos^ primary oncology patients with HPV16 integration (*N* = 9)	ccfHPV^neg^ primary oncology patients with HPV16 integration (*N* = 4)	*P*‐value
Tumor size	***n* (%)**	***n* (%)**	0.86^a^
≥ 20 mm < 30 mm	1 (11.1)	0 (0.0)	
≥ 40 mm	8 (88.9)	4 (100.0)	
Mean ± SD	48.4 ± 12.9	49.8 ± 9.8	
Median (IQR)	45 (10.0)	49.5 (19.3)	
FIGO 2018	***n* (%)**	***n* (%)**	0.05^b^
IB3	1 (11.1)	0 (0.0)	
IIB	2 (22.2)	3 (75.0)	
IIIC1	6 (66.7)	0 (0.0)	
IVA	0 (0.0)	1 (25.0)	

a Unpaired two‐sided *t*‐test.

b Fisher's exact test.

## 4 Discussion

This study examined the performance of our targeted NGS HPV genotyping panel for detection of ccfHPV DNA in plasma from 139 CC patients. Results on ccfHPV DNA were used to assess the value of ccfHPV DNA as a marker for disease severity. ccfHPV DNA was detected in 66 (47.5%) patients: four primary surgery patients (8.0%); eight (36.4%) primary surgery + adjuvant oncology patients, four (33.3%) intermediate‐risk patients (*P =* 0.04) and four (40.0%) high‐risk patients (*P =* 0.02); and 54 (80.6%) primary oncology patients (*P =* 0.00). Furthermore, in agreement with previous reports [17, 24, 25, 45, 46], ccfHPV analyses of plasma from 25 CIN3 patients and 15 healthy individuals were all negative, indicating specificity only for CCs. Overall, the detection rates in CC patients were higher than in our previous proof‐of‐concept study using ddPCR for ccfHPV DNA detection [26], which may be explained by the different qualities of the two assays; the ddPCR assay provides a quantitative measure of ccfHPV DNA based on one primer set and corresponding probe for HPV16 and 18, respectively, whereas the NGS panel includes multiple primer sets and thus covers several areas of the HPV genome for the two genotypes, ensuring that if genetic changes or integration have occurred in one or more regions of the HPV genome, ccfHPV DNA would still be detected.

Including only patients from the primary surgery and primary oncology subgroup (*n* = 117) and applying the previously mentioned assumptions on true and false positives and true and false negatives, our NGS HPV genotyping panel showed a sensitivity of 80.6%, a specificity of 92.0%, a PPV of 93.1%, and a NPV of 78.9%, showing that ccfHPV DNA is a very promising marker to identify patients requiring primary oncological therapy.

As previously described, detection of ctDNA is widely used for detecting and monitoring disease in various cancer types [13, 15, 16], and with HPV being a critical step in the carcinogenesis of HPV‐related CC, these patients are an ideal model to detect ctDNA by detecting HPV DNA. In previous studies on ccfHPV DNA in patients with HPV‐related diseases, detection rates vary between 31% and 100% [17, 18, 19, 20, 21, 22, 23, 24, 25]. Possible explanations for these inconclusive results are the use of different analysis methods, a great variation in the number of patients included and importantly, in patient characteristics, for example, disease stage. Furthermore, there is a large variation in the determination of a reliable cut‐off for ccfHPV DNA positivity, especially because some studies fail to include negative control samples. In agreement with similar studies [17, 23, 25], our recent study on ccfHPV DNA in CC patients [26] found that ccfHPV DNA detection and quantity are closely related to tumor size and disease stage and may be a promising tool to establish tumor burden in patients with LACC. As in these prior studies, results from this current study show that ccfHPV DNA is only detectable in very few patients referred for primary surgery, whereas for patients referred for primary surgery + adjuvant oncological therapy and especially patients referred for primary oncological therapy, ccfHPV DNA is frequently detected. Because of the much debated usefulness of Sedlis criteria for intermediate‐risk patients [7, 8, 9], we examined the qualitative detection of ccfHPV DNA for patients in the primary oncology + adjuvant oncology subgroup deeper and found that four (33.3%) of the 12 intermediate‐risk patients and four (40.0%) of the 12 high‐risk patients were ccfHPV^pos^ at diagnosis. Thus, for intermediate‐risk patients, it is plausible that compared to the ccfHPV^neg^ patients, the clinical characteristics for these specific patients are different, for example, tumor size, LVSI, and DOI, and these parameters may influence whether ccfHPV DNA is present or not. Due to the low number of patients in this study, we did not examine these possible differences. However, since both prior studies and the current study have established that ccfHPV DNA is a valid marker for identifying patients in need of primary oncological therapy, it is plausible that the ccfHPV^pos^ intermediate‐risk patients may be the patients in this subgroup who benefitted from adjuvant therapy, whereas the remaining eight patients did not. However, to examine the validity of the use of ccfHPV DNA for a better treatment stratification of patients in the intermediate‐risk group, outcome data on local control, disease and overall survival, and a larger patient cohort are needed.

Based on the well‐known impact of high‐risk factors on disease recurrence in high‐risk patients [5, 6], the rather low detection rate in these patients seems surprising. Similarly, the results on ccfHPV DNA‐negative primary oncology patients are unexpected, and these results explain the only moderate sensitivity of 80.6% and NPV of 78.9% in this study. However, similarly to our findings, previous studies on ctDNA of somatic mutations in different cancer types have shown that around 70–88% of patients with metastatic disease have detectable levels of ctDNA [47, 48], even though tissue analyses of these patients have shown positivity for specific mutations. ccfHPV DNA detection and quantity are closely related to disease severity [17, 23, 25, 26] and also HPV genotype [18, 23], the last‐mentioned possibly due to differences in integration frequency according to HPV genotype. Thus, we explored the primary oncology subgroup more thoroughly in regard to different clinical parameters as well as HPV16 integration status, which may be factors explaining ccfHPV DNA negatives in both high‐risk and primary oncology patients. Overall, when comparing both histology (SCC vs. AC/ASC), HPV genotype (HPV18/45 vs. all other HPV genotyping results), tumor size, and FIGO stage between ccfHPV^pos^ and ccfHPV^neg^ primary oncology patients, we found no significant differences (*P* = 1.00, *P* = 0.26, *P* = 0.61, and *P* = 0.64, respectively; Table 3). Especially findings on no difference according to either tumor size or disease stage conflict with both our findings that significantly more primary oncology patients and primary surgery plus adjuvant oncology patients are ccfHPV DNA positive than primary surgery patients (Table 2) as well as previous findings showing that ccfHPV DNA level is correlated to disease severity [17, 18, 23, 25, 26]. These findings may therefore very likely be caused by the limited number of patients in this study. However, other factors may also explain the ccfHPV^neg^ findings in primary oncology patients. The findings of no difference according to HPV genotype suggest no impact of integration on ccfHPV DNA detection. Nevertheless, even if not statistically significant, our genotyping results showed that four (30.8%) of ccfHPV^neg^ primary oncology patients and only nine (16.7%) ccfHPV^pos^ primary oncology patients had HPV18/45‐related CC (Table 3), and since other HPV genotypes than HPV18 and HPV45 may also integrate in over 50% of cases [30, 40, 41] and thus represent some of the remaining ccfHPV^neg^ patients, an impact of integration status and thus low viral load should not be fully rejected. Besides affecting viral load [30, 31], integration status also seem to affect prognosis [30, 31, 32, 33], and studies have shown that cancer patients with HPV integration have significantly shortened disease‐free survival (DFS) compared to patients with non‐integrated HPV [30, 31, 33]. Similarly, results from the study by Kiseleva et al. [32] suggest that HPV16 DNA integration is an independent factor for predicting clinical outcome of LACC and can serve as an effective criterion for the individual choice of treatment tactics for the patients. As previously described, HPV integration occurs due to disruption of specific genes in the HPV genome, most frequently the E1/E2 genes, which causes constitutive expression of the E6/E7 oncoproteins [30]. Taken together, one would expect higher steady‐state levels of E6/E7 transcripts in integrated CCs because of virus integration. However, previous findings have shown that HPV integration *per se* does not result in an increase in E6/E7 oncogene expression [30, 31, 49, 50, 51, 52]. Thus, the mechanism behind the worsened prognosis in integrated patients may be more complex than viral integration and E1/E2 disruption. Findings from previous studies suggest that epigenetic factors, that is, methylation of HPV [52] and genetic factors, that is, down‐modulation of Notch1 [53] or activation of the proto‐oncogene MYC [54], affect E6/E7 expression and thus these factors are likely to play an important role in HPV‐induced carcinogenesis.

In our study, integration status was successfully assessed in cervical tissue DNA from the 67 CC patients from the study cohort with HPV16‐related disease by our recently developed NGS HPV16 panel. Complete integration was found in 19 (28.4%) patients, and not surprisingly, we found that significantly more ccfHPV^neg^ primary oncology patients than ccfHPV^pos^ patients had complete integration (*P* = 0.04) (Table 4), probably showing that ccfHPV DNA detection is influenced by HPV integration status. Thus, the lack of episomal HPV DNA in the HPV16 integrated patients may be the cause of non‐detectable ccfHPV DNA (ccfHPV^neg^) in some primary oncology patients and possibly in some high‐risk patients (ccfHPV^neg^) as well. This may be attributed to low DNA viral load, as well as the fact that HPV DNA from cancer cells with integrated HPV is probably only released as a consequence of cell death. Contrary, cancer cells from patients with non‐integrated HPV have a high DNA viral load and harbor free and intact HPV genomes. It seems plausible that virus DNA can be released to the general circulation both with and without preceding cell death, thus increasing the rate of detection of ccfHPV DNA. These findings are in line with previous data showing that ccfHPV DNA positivity is influenced by HPV genotype [18, 23], and interestingly, Cabel et al. [23] found that ccfHPV DNA positivity for HPV18 is significantly lower (20%, *n* = 2/10) than for HPV16 (77%, *n* = 27/35), which is also supported by findings from other studies [18, 24].

When assessing potential differences in disease severity in primary oncology patients with HPV16 integration, we found that ccfHPV^pos^ patients had higher disease stages than ccfHPV^neg^ patients (*P* = 0.05) (Table 5), which may suggest that detection of ccfHPV DNA in patients with integrated HPV is influenced by disease stage, for example, lymph node status or spread of the tumor to adjacent or distant organs. To examine this deeper, a larger cohort is needed.

## 5 Conclusions

In conclusion, our study showed that our NGS HPV genotyping panel is useful for ccfHPV DNA detection, that our NGS HPV16 panel may be used for assessing HPV16 integration status, and that ccfHPV DNA represents a promising marker for identifying patients requiring primary oncological therapy. Furthermore, it may add to the currently available Sedlis criteria used to guide adjuvant therapy in intermediate‐risk patients. However, detection of ccfHPV DNA seems to be influenced by HPV integration status and disease stage, and thus, these factors need to be considered in ccfHPV^neg^ patients. Future studies should further examine the validity of the use of ccfHPV DNA for a better treatment stratification of intermediate‐risk patients but also the use of ccfHPV DNA for establishing treatment response and monitoring patients after treatment.

## Conflict of interest

The authors declare no conflict of interest.

## Author contributions

SB, TS, JB, MS, KF, and JCL formulated the hypothesis and design of the study. SB obtained the financial support acquired for the project leading to this publication. SB administrated the study, and TS, JB, MS, BSS, JEB, G‐BN, JCL, KF, MLS, and SL supervised during the course of the study. KF and G‐BN assisted in patient inclusion. JEB and BSS performed laboratory procedures. Formal analyses of NGS data were performed by SB and supervised by TS, and MS Statistical considerations and analyses were performed by SB. SB completed the first draft of the manuscript, which was reviewed and edited by TS, JB, MS, BSS, JEB, G‐BN, JCL, KF, MLS and SL. All authors agree to be accountable for all aspects of the work and have read and agreed to the published version of the manuscript.

### Peer review

The peer review history for this article is available at https://www.webofscience.com/api/gateway/wos/peer‐review/10.1002/1878‐0261.13538.

## Supporting information


**Table S1.** Clinical and biological data on the study cohort.
**Table S2.** Background reads in cases with non‐HPV16‐related cancer.
**Table S3.** Qualitative assessment of ccfHPV DNA using NGS HPV genotyping on 25 CIN3 patients and 15 negative controls.
**Table S4.** ccfHPV DNA‐positive patients from the case cohort with results closest cut‐off for HPV positivity.
**Table S5.** ccfHPV^neg^ patients from the case cohort with any detectable HPV reads.
**Table S6.** Qualitative assessment of tissue HPV DNA using NGS HPV16 panel on ten cases with non‐HPV16‐related cervical cancer.
**Table S7.** Qualitative assessment of ccfHPV DNA.
**Table S8.** Analyses of tissue samples from the 67 cases positive for HPV16 (single genotype infection) in tumor tissue using NGS HPV16 panel.
**Table S9.** Disease burden in primary oncology patients according to HPV16 integration status.

## Data Availability

All datasets generated during this study are available in the supplementary material document (including Table S1: Clinical and biological data on the study cohort, Table S2. Background reads in cases with non‐HPV16‐related cancer (*N* = 63), Table S3. Qualitative assessment of ccfHPV DNA using NGS HPV genotyping on 25 CIN3 patients and 15 negative controls, Table S4. ccfHPV DNA‐positive patients from the case cohort with results closest cut‐off for HPV positivity (*n* = 14), Table S5. ccfHPV^neg^ patients from the case cohort with any detectable HPV reads (*n* = 16), Table S6. Qualitative assessment of tissue HPV DNA using NGS HPV16 panel on 10 cases with non‐HPV16‐related CC (*n* = 10), Table S7. Qualitative assessment of ccfHPV DNA, Table S8: Analyses of tissue samples from the 67 cases positive for HPV16 (single genotype infection) in tumor tissue using NGS HPV16 panel, and Table S9. Disease burden in primary oncology patients according to HPV16 integration status).
