# Supplementary material for: Circulating cell‐free HPV DNA is a strong marker for disease severity in cervical cancer
Source: Mol Oncol. 2023 Nov 1;18(5):1231–44. doi: 10.1002/1878-0261.13538 (PMC11076984; doi:10.1002/1878-0261.13538)
Supplement: Supplementary file 1 — Table S1. Clinical and biological data on the study cohort. Table S2. Background reads in cases with non‐HPV16‐related cancer. Table S3. Qualitative assessment of ccfHPV DNA using NGS HPV genotyping on 25 CIN3 patients and 15 negative controls. Table S4. ccfHPV DNA‐positive patients from the case cohort with results closest cut‐off for HPV positivity. Table S5. ccfHPVneg patients from the case cohort with any detectable HPV reads. Table S6. Qualitative assessment of tissue HPV DNA using NGS HPV16 panel on ten cases with non‐HPV16‐related cervical cancer. Table S7. Qualitative assessment of ccfHPV DNA. Table S8. Analyses of tissue samples from the 67 cases positive for HPV16 (single genotype infection) in tumor tissue using NGS HPV16 panel. Table S9. Disease burden in primary oncology patients according to HPV16 integration status. [file MOL2-18-1231-s001.docx]

**Supplementary material for “Circulating cell-free HPV DNA is a strong marker for disease severity in cervical cancer.**

## **Sara Bønløkke ^1, 2^**^,^ **, Torben Steiniche ^1, 2^, Boe Sandahl Sorensen ^1, 3^, Gitte-Bettina Nyvang ^4^, Jacob Christian Lindegaard ^5^, Jan Blaakær ^6, 7^ , Jesper Bertelsen ^2^, Katrine Fuglsang ^8^, Mikael Lenz Strube ^9^, Suzan Lenz ^10^ and Magnus Stougaard ^1, 2^**

| Table S1: Clinical and biological data on the study cohort | | | | | | | | | |
| --- | --- | --- | --- | --- | --- | --- | --- | --- | --- |
| *Primary surgery subgroup (n=50) ^1^* | | | | | | | | | |
| Pt no. | Age | Histology^2^ | FIGO 2018^3^ | T-score^4^ | Tumor size (mm)^5^ | Treatment^6^ | Histologically re-staged^7^ | HPV tissue INNOLiPA^8^ | HPV tissue NGS genotyping panel^9^ |
| 1 | 46 | ASC | IB2 | 2 | 20 | Rad hys+PL+BSO |  | HPV18 | HPV18 |
| 2 | 36 | AC | IB1 | 1 | 10 | Rad hys+PL+BSO |  | HPV18 | HPV18 |
| 3 | 44 | ASC | IB1 | 2 | 26 | Rad hys+PL+BSO | IB2 | HPV18 | HPV18 |
| 4 | 36 | AC | IB1 | 2 | 24 | Rad hys+SLN+BSO | IB2 | HPV16 | HPV16 |
| 5 | 41 | SCC | IB1 | 1 | 8 | Rad hys+SLN+BSO |  | HPV16 | HPV16 |
| 6 | 47 | SCC | IA2 | 0,5 | 7,5 | Rad hys+SLN+BSO |  | HPV16 | HPV16 |
| 7 | 43 | SCC | IA2 | 0,5 | 10 | Rad hys+SLN+BSO |  | HPV16 | HPV16 |
| 8 | 57 | SCC | IB2 | 2 | 22 | Rad hys+SLN+BSO |  | HPV16 | HPV16 |
| 9 | 73 | SCC | IB2 | 2 | 10 | Rad hys+SLN+BSO |  | HPV16 | HPV16 |
| 10 | 51 | SCC | IA2 | 0,5 | 9 | Simple hys+PL+BSO |  | HPV16 | HPV16 |
| 12 | 72 | AC | IA2 | 0,5 | 7,2 | TLH+PL+BSO |  | HPV16 | HPV16 |
| 13 | 40 | AC | IB1 | 1 | 19 | Rad hys+SLN+BSO |  | HPV16 | HPV16 |
| 14 | 30 | SCC | IA2 | 0,5 | 14 | Rad hys+SLN+BSO |  | HPV18 | HPV18 |
| 15 | 41 | AC | IB1 | 1 | 8,1 | Rad hys+SLN+BSO |  | HPV18 | HPV18 |
| 16 | 47 | SCC | IA2 | 0,5 | 8 | Rad hys+SLN+BSO |  | HPV18 | HPV18 |
| 17 | 43 | SCC | IB2 | 1 | 12 | Rad hys+SLN+BSO | IB1 | HPV16 | HPV16 |
| 18 | 33 | SCC | IA1 | 0,25 | 17 | Collum amputation+SLN |  | HPV16 | HPV16 |
| 19 | 44 | AC | IA2 | 0,5 | 12 | Rad hys+SLN+BSO |  | HPV18 | HPV18+HPV45 |
| 20 | 83 | SCC | IB1 | 1 | 10 | Rad hys+SLN+BSO |  | HPV16 | HPV16+HPV33 |
| 21 | 39 | AC | IB1 | 1 | 18 | Rad hys+SLN+PL+BSO+omenectomy |  | HPV18 | HPV18 |
| 22 | 44 | AC | IB2 | 2 | 22 | Rad hys+SLN+BSO |  | HPV16 | HPV16 |
| 23 | 34 | SCC | IB2 | 2 | 20 | Rad hys+SLN+BSO |  | HPV16 | HPV16 |
| 24 | 50 | AC | IA2 | 0,5 | 8 | Rad hys+SLN+BSO |  | HPV16 | HPV16 |
| 25 | 66 | AC | IA2 | 0,5 | 10 | Rad hys+SLN+BSO |  | HPV16 | HPV16 |
| 26 | 33 | AC | IA2 | 0,5 | 13 | Rad hys+SLN+bilat salpingectomy |  | HPV16 | HPV16 |
| 27 | 63 | AC | IB1 | 2 | 28 | Rad hys+SLN+BSO | IB2 | HPV18 | HPV18 |
| 28 | 40 | AC | IB2 | 2 | 24 | Rad hys+SLN+BSO |  | HPV16 | HPV16 |
| 29 | 33 | SCC | IB1 | 1 | 18 | Rad hys+SLN+BSO |  | HPV16 | HPV16 |
| 30 | 56 | SCC | IB1 | 2 | 23 | Rad hys+SLN+BSO | IB2 | HPV16 | HPV16 |
| 61 | 41 | ASC | IB1 | 1 | 12 | Rad hys+SLN+BSO |  | HPV45 | HPV45 |
| 62 | 56 | SCC | IB2 | 2 | 30 | Rad hys+SLN+BSO |  | HPV39 | HPV39 |
| 63 | 59 | SCC | IB1 | 1 | 9 | Rad hys+SLN+BSO |  | HPV68 | HPV68 |
| 64 | 46 | SCC | IA2 | 0.5 | 8,4 | TLH + SLN+vaginal collar+BSO |  | HPV16 | HPV16 |
| 65 | 37 | ASC | IA2 | 0.5 | 13,2 | Rad hys+SLN |  | HPV18+HPV66 | HPV18 |
| 66 | 36 | SCC | IB1 | 1 | 9 | Rad hys+SLN+BSO |  | HPV39 | HPV39 |
| 67 | 57 | SCC | IB1 | 0.5 | 22 | Rad hys+SLN |  | HPV31 | HPV31 |
| 68 | 33 | ASC | IB2 | 2 | 29 | Rad hys+SLN+BSO |  | HPV45 | HPV45 |
| 69 | 30 | SCC | IB2 | 1 | 19 | Rad hys+SLN+BSO | IB1 | HPV33 | HPV33 |
| 70 | 36 | SCC | IB2 | 2 | 24 | Rad hys+SLN+BSO |  | HPV16+HPV33 | HPV16+HPV33 |
| 71 | 47 | SCC | IB3 | 3 | 43 | Rad hys+SLN+PL |  | HPV45 | HPV45 |
| 72 | 44 | AC | IB1 | 1 | 19 | Rad hys+SLN+BSO |  | HPV45 | HPV45 |
| 73 | 31 | SCC | IB2 | 2 | 22 | Rad hys+SLN+BSO |  | HPV16 | HPV16 |
| 74 | 37 | SCC | IB1 | 1 | 19 | TLH+SLN+BSO |  | HPV31 | HPV31 |
| 75 | 59 | SCC | IB1 | 1 | 6 | Rad hys+SLN+salpingooophorectomy dxt |  | HPV16 | HPV16 |
| 76 | 60 | SCC | IB2 | 3 | 48 | Rad hys+SLN+BSO | IB3 | HPV16 | HPV16+HPV67 |
| 77 | 36 | AC | IB1 | 1 | 17 | Rad hys+SLN+BSO |  | HPV18 | HPV18 |
| 78 | 39 | AC | IB2 | 2 | 35 | Rad hys+SLN+BSO |  | HPV18 | HPV18 |
| 79 | 34 | SCC | IB1 | 0.5 | 15 | Rad hys+SLN+BSO | IA1 | HPV16 | HPV16 |
| 80 | 51 | SCC | IA2 | 0.5 | 11 | Rad hys+SLN+BSO |  | HPV45 | HPV45 |
| 81 | 52 | SCC | IA2 | 0.5 | 10 | Rad hys+SLN+BSO |  | HPV45+HPV16 | HPV45+HPV16 |
| *Primary surgery + adjuvant oncology subgroup (n=22)* | | | | | | | | | |
| Pt no. | Age | Histology | FIGO 2018 | T-score | Tumor size (mm) | Treatment | Histologically re-staged | HPV tissue INNOLiPA | HPV tissue NGS genotyping panel |
| 11 | 36 | SCC | IB1 | 2 | 25 | Rad hys+SLN+BSO^11^ | IB2^12^ | HPV16 | HPV16 |
| 82 | 45 | AC | IA2 | 2 | 22 | Rad hys+PL+BSO followed by EBRT+Cis | IB2^13^ | HPV16 | HPV16 |
| 83 | 40 | AC | IB2 | 2 | 21 | Rad hys+PL+BSO followed by EBRT+Cis | IIIC1^13^ | HPV18 | HPV18 |
| 84 | 42 | ASC | IB2 | 2 | 24 | Rad hys+PL+BSO followed by EBRT+Cis | ^12^ | HPV45 | HPV45 |
| 85 | 29 | SCC | IB2 | 2 | 38 | Rad hys+PL+BSO followed by EBRT+Cis | ^12^ | HPV16 | HPV16 |
| 86 | 37 | AC | IB2 | 2 | 32 | Rad hys+SLN+BSO followed by EBRT+Cis | ^12^ | HPV18 | HPV18 |
| 87 | 60 | SCC | IB2 | 2 | 33 | Rad hys+PL+BSO followed by EBRT+Cis | ^12^ | HPV45 | HPV45 |
| 88 | 66 | SCC | IB2 | 2 | 22 | Rad hys+SLN followed by EBRT+Cis | IIIC1^13^ | HPV16+HPV70 | HPV16+HPV70 |
| 89 | 52 | SCC | IB1 | 2 | 25 | Rad hys+SLN+BSO followed by EBRT+Cis | IB2^12^ | HPV31 | HPV31 |
| 90 | 43 | AC | IB2 | 2 | 43 | Rad hys+SLN+BSO followed by EBRT+Cis | IIIC1^13^ | HPV18 | HPV18 |
| 91 | 35 | ASC | IIA1 | 3 | 39 | Rad hys+SLN+BSO followed by EBRT+Cis | ^12^ | HPV18 | HPV18 |
| 92 | 67 | SCC | IIA1 | 3 | 24 | Rad hys+SLN+BSO+ vaginal collar followed by EBRT+Cis | IIIC1^13^ | HPV33 | HPV33 |
| 93 | 58 | SCC | IIA1 | 3 | 22 | Rad hys+SLN+BSO followed by EBRT+Cis | ^12^ | HPV16 | HPV16 |
| 94 | 70 | SCC | IB2 | 2 | 22 | Rad hys+SLN+BSO followed by EBRT+vaginal BT | IIIC1^13^ | HPV35 | HPV35 |
| 95 | 43 | SCC | IB2 | 2 | 21 | Rad hys+SLN+BSO followed by EBRT+Cis | IIIC1^13^ | HPV16 | HPV16 |
| 96 | 39 | AC | IB2 | 2 | 20 | Rad hys+SLN+BSO followed by EBRT+Cis | IIIC1^13^ | HPV16+HPV18 | HPV16+HPV18 |
| 97 | 40 | SCC | IIA1 | 3 | 20 | Rad hys+SLN+BSO followed by EBRT+Cis | IIIC2^13^ | HPV16 | HPV16 |
| 98 | 33 | SCC | IB2 | 2 | 23 | Rad hys+SLN+BSO followed by EBRT+Cis | IIIC1^13^ | HPV73 | HPV73 |
| 99 | 64 | SCC | IB2 | 2 | 29 | Rad hys+SLN+BSO followed by EBRT+Cis | ^12^ | HPV18 | HPV18 |
| 100 | 55 | SCC | IB2 | 2 | 35 | Rad hys+SLN+BSO followed by EBRT+Cis | IIIC1^13^ | HPV16 | HPV16 |
| 101 | 40 | SCC | IB1 | 2 | 20 | Rad hys+SLN+BSO^11^ | IB2^12^ | HPV33 | HPV33 |
| 102 | 76 | SCC | IB3 | 3 | 43 | Rad hys+SLN+PL^11^ | ^12^ | HPV positive | HPV30 |
| *Primary oncology subgroup (n=67)^10^* | | | | | | | | | |
| Pt no. | Age | Histology | FIGO 2018 | T-score | Tumor size (mm) | Treatment | Histologically re-staged | HPV tissue INNOLiPA | HPV tissue NGS genotyping panel |
| 31 | 60 | AC | llB | 3 | 35 | EBRT+BT+Cis | N/A^14^ | HPV16 | HPV16 |
| 32 | 72 | SCC | llB | 3 | 40 | EBRT+BT+Cis | N/A | HPV16 | HPV16 |
| 33 | 56 | SCC | llB | 4 | 50 | EBRT+BT+Cis | N/A | HPV16 | HPV16 |
| 34 | 49 | SCC | IIIC1 | 5 | 40 | EBRT+BT+Cis | N/A | HPV16 | HPV16 |
| 35 | 53 | SCC | IIB | 4 | 40 | EBRT+BT+Cis | N/A | HPV16 | HPV16 |
| 36 | 50 | SCC | IIB | 6 | 50 | EBRT+BT+Cis | N/A | HPV16 | HPV16 |
| 37 | 61 | SCC | IIIC1 | 9 | 60 | EBRT+BT+Cis | N/A | HPV16 | HPV16 |
| 38 | 85 | SCC | IIB | 4 | 50 | EBRT+BT | N/A | HPV16 | HPV16 |
| 39 | 40 | SCC | IIB | 3 | 28 | EBRT+BT+Cis | N/A | HPV16 | HPV16 |
| 40 | 42 | SCC | IIIC1 | 3 | 35 | EBRT+BT+Cis | N/A | HPV18 | HPV18 |
| 41 | 51 | SCC | IIB | 7 | 65 | EBRT+BT+Cis | N/A | HPV16 | HPV16 |
| 42 | 41 | SCC | IIIC1 | 7 | 55 | EBRT+BT+Cis | N/A | HPV16 | HPV16 |
| 43 | 35 | SCC | IIIC1 | 2 | 24 | EBRT+BT+Cis | N/A | HPV16 | HPV16 |
| 44 | 46 | SCC | IIIC1 | 3 | 35 | EBRT+BT+Cis | N/A | HPV18 | HPV18 |
| 45 | 55 | AC | llB | 6 | 55 | EBRT+BT+Cis | N/A | HPV16 | HPV16 |
| 46 | 59 | SCC | lVA | 17 | 59 | Neoadj Taxol/Topotecan/Avastin followed by EBRT+BT | N/A | HPV16 | HPV16 |
| 47 | 30 | SCC | IIIC1 | 5 | 30 | EBRT+BT+Cis | N/A | HPV16 | HPV16 |
| 48 | 77 | SCC | IIIC1 | 5 | 45 | EBRT+BT+Cis | N/A | HPV16 | HPV16 |
| 49 | 46 | SCC | IIIC1 | 5 | 50 | EBRT+BT+Cis | N/A | HPV16 | HPV16 |
| 50 | 52 | AC | IIB | 3 | 40 | EBRT+BT+Cis | N/A | HPV16 | HPV16 |
| 51 | 43 | SCC | IIB | 3 | 42 | EBRT+BT+Cis | N/A | HPV16 | HPV16 |
| 52 | 57 | SCC | IIIC1 | 5 | 40 | EBRT+BT+Cis | N/A | HPV16 | HPV16 |
| 53 | 27 | SCC | IIIC2 | 10 | 70 | EBRT+BT+Cis | N/A | HPV16 | HPV16 |
| 54 | 55 | SCC | IIB | 7 | 45 | EBRT+BT+Cis | N/A | HPV16 | HPV16 |
| 55 | 53 | SCC | IB3 | 3 | 40 | EBRT+BT+Cis | N/A | HPV16 | HPV16 |
| 56 | 40 | SCC | IIIC1 | 9 | 100 | EBRT+BT+Cis | N/A | HPV16 | HPV16 |
| 57 | 41 | SCC | IIIC1 | 3 | 70 | EBRT+BT+Cis | N/A | HPV16 | HPV16 |
| 58 | 32 | SCC | IB3 | 3 | 45 | EBRT+BT+Cis | N/A | HPV16 | HPV16 |
| 59 | 57 | SCC | IIIC1 | 11 | 80 | EBRT+BT+Cis | N/A | HPV18 | HPV18 |
| 60 | 55 | AC | IIIC2 | 10 | 100 | Neoadjuvant Cis followed by EBRT+BT+Cis | N/A | HPV16 | HPV16 |
| 103 | 77 | SCC | IIIB | 15 | 120 | EBRT+BT+Cis |  | HPV58 | HPV58 |
| 104 | 60 | SCC | IIIC1 | 12 | 50 | EBRT+BT+Cis |  | HPV45+HPV52 | HPV45 |
| 105 | 74 | SCC | IIB | 3 | 30 | EBRT+BT+Cis |  | HPV33 | HPV33 |
| 106 | 82 | SCC | IIB | 5 | 30 | EBRT+BT |  | HPV33 | HPV30+HPV33 |
| 107 | 50 | SCC | IIB | 5 | 40 | EBRT+BT+Cis |  | HPV16+HPV58 | HPV16 |
| 108 | 61 | SCC | IIB | 5 | 35 | EBRT+BT+Cis |  | HPV11 | HPV11 |
| 109 | 81 | SCC | IIB | 5 | 45 | EBRT+BT |  | HPV39 | HPV39 |
| 110 | 81 | SCC | IIB | 10 | 40 | EBRT+BT |  | HPV33 | HPV33 |
| 111 | 59 | SCC | IIIC1 | 4 | 35 | EBRT+BT+Cis |  | HPV45 | HPV45 |
| 112 | 32 | SCC | IIIC1 | 5 | 45 | EBRT+BT+Cis |  | HPV45 | HPV45 |
| 113 | 34 | SCC | IIIC1 | 9 | 120 | EBRT |  | HPV45+HPV16 | HPV45+HPV16 |
| 114 | 64 | SCC | IIB | 6 | 75 | EBRT+BT |  | HPV33 | HPV33 |
| 115 | 52 | SCC | IIB | 5 | 35 | EBRT+BT+Cis |  | HPV52 | HPV52 |
| 116 | 70 | SCC | IVA | 6 | 65 | EBRT+BT+Cis |  | HPV16 | HPV16 |
| 117 | 87 | SCC | IIB | 5 | 45 | EBRT+BT |  | HPV45 | HPV45 |
| 118 | 34 | SCC | IIB | 7 | 60 | EBRT+BT+Cis |  | HPV16 | HPV16 |
| 119 | 69 | SCC | IIB | 4 | 60 | EBRT+BT |  | HPV16 | HPV16 |
| 120 | 78 | SCC | IIIC1 | 6 | 40 | EBRT+BT+external boost |  | HPV16 | HPV16 |
| 121 | 54 | SCC | IIIC1 | 3 | 30 | EBRT+BT+Cis |  | HPV45 | HPV45 |
| 122 | 82 | SCC | IIIC2 | 7 | 56 | EBRT+BT+Cis |  | HPV18 | HPV18 |
| 123 | 54 | SCC | IIIC1 | 8 | 60 | EBRT+BT |  | HPV45 | HPV45 |
| 124 | 71 | SCC | IIB | 6 | 60 | EBRT+BT+Cis |  | HPV31 | HPV31 |
| 125 | 65 | SCC | IIIC1 | 7 | 60 | EBRT+BT+Cis |  | HPV70 | HPV70 |
| 126 | 36 | SCC | IIIC1 | 4 | 50 | EBRT+BT+Cis |  | HPV16 | HPV16 |
| 127 | 60 | SCC | IIIC1 | 13 | 80 | EBRT+BT+Cis |  | HPV16 | HPV16 |
| 128 | 45 | SCC | IIB | 6 | 45 | EBRT+BT+Cis |  | HPV18 | HPV18 |
| 129 | 71 | SCC | IIIC1 | 11 | 63 | EBRT+BT+Cis |  | HPV16 | HPV16 |
| 130 | 44 | SCC | IIIC1 | 4 | 38 | EBRT+BT+Cis |  | HPV16 | HPV16 |
| 131 | 27 | SCC | IIIC1 | 5 | 70 | EBRT+BT+Cis |  | HPV31 | HPV31 |
| 132 | 79 | SCC | IIIC1 | 6 | 50 | EBRT+BT+Cis |  | HPV31 | HPV31 |
| 133 | 55 | SCC | IIIC1 | 9 | 80 | EBRT+BT+Cis |  | HPV18+HPV16 | HPV18+HPV16 |
| 134 | 50 | SCC | IIIC1 | 5 | 35 | EBRT+BT+Cis |  | HPV33 | HPV33 |
| 135 | 37 | SCC | IIB | 4 | 60 | EBRT+BT+Cis |  | HPV16+HPV45 | HPV45 |
| 136 | 44 | SCC | IB3 | 3 | 45 | EBRT+BT+Cis |  | HPV45 | HPV45 |
| 137 | 41 | AC | IIIC1 | 3 | 42 | EBRT+BT+Cis |  | HPV45 | HPV45+HPV16 |
| 138 | 47 | SCC | IIIC1 | 5 | 55 | EBRT+BT+Cis |  | HPV18 | HPV18 |
| 139 | 44 | SCC | IIIC1 | 3 | 75 | EBRT+BT+Cis |  | HPV16 | HPV16 |

^1^ Primary surgery patient 1-30 is included in the proof-of-concept study in Bonlokke, S., et al., *The Diagnostic Value of Circulating Cell-Free HPV DNA in Plasma from Cervical Cancer Patients.* Cells, 2022. **11**(14).). For patient number 11 however, we later discovered that she was actually categorized as having intermediar risk factors after surgery but that she had refused to receive adjuvant radiochemotherapy. For the present study, she has therefore been moved to the correct primary surgery+adjuvant oncology subgroup.

^2^ Abbreviations: Squamous cell carcinoma (SCC), adenocarcinoma (AC), adenosquamous carcinoma (ASC)

^3^ Disease stage according to FIGO 2018 (Bhatla, N., et al., *Revised FIGO staging for carcinoma of the cervix uteri.* Int J Gynaecol Obstet, 2019. 145(1): p. 129-135.). For patients having been re-staged after surgery, the re-staged stage is the one listed.

^4^ T-score according to Lindegaard, J.C., et al., *Prognostic implications of uterine cervical cancer regression during chemoradiation evaluated by the T-score in the multicenter XXX study.* Int J Radiat Oncol Biol Phys, 2022. The scoring system is developed for cervical cancer patients with stage IB-IVB disease, giving patients with stage IB1, IB2, and IB3 a T-score of 1, 2, and 3 respectively. Thus, for patients with stage IA1 and IA2 disease, we made a presumption that stage IA1 equals a T-score of 0.25 and stage IA2 equals a T-score of 0.5. For patients having been re-staged after surgery, the re-staged stage is the one used to determine T-score.

^5^ Larges diameter of tumor. For the primary surgery subgroup and the primary surgery+adjuvant oncology subgroup, tumor size was evaluated pathologically *after* surgery based on the removed tissue. For the primary oncology subgroup, tumor size was evaluated based on clinical examination or magnetic resonance imaging (MRI) prior to treatment.

^6^ Treatment abbreviations:

- Rad hys = Radical hysterectomy.
- PL = Pelvic lymphadenectomy.
- BSO = Bilateral salpingo-oophorectomy.
- SLN = Sentinel lymph node (SLN) removal
- Sim hys = Simple hysterectomy.
- TLH = Total laparoscopic hysterectomy
- Vaginal collar = Removal of vaginal collar.
- EBRT = External beam radiation therapy
- BT = Brachytherapy.
- Cis = Concomitant weekly cisplatin intravenously.

^7^ Pathological re-staging *after* surgery based on histopathological detection of intermediar (tumor size > 3 cm and depth of stromal invasion (DSI) > 2/3; tumor size > 2 cm and DSI > 1/3 and lymphovascular space invasion (LVSI)) or high-risk (lymph node metastasis, parametrial invasion, and positive resection margin) factors. See See Bhatla, N., et al., *Revised FIGO staging for carcinoma of the cervix uteri.* Int J Gynaecol Obstet, 2019. **145**(1): p. 129-135.

^8^ Cervical tissue biopsy tested with INNOLiPA® Genotyping Extra II (Fujirebio)

^9^ Cervical tissue biopsy tested with targeted Next Generation Sequencing panel described in Andersen K, Holm K, Tranberg M, Pedersen CL, Bonlokke S, Steiniche T, et al. Targeted Next Generation Sequencing for Human Papillomavirus Genotyping in Cervical Liquid-Based Cytology Samples. Cancers (Basel). 2022;14(3).

^10^ Primary oncology patient 31-60 is included in the proof of concept study by Bonlokke, S., et al., *The Diagnostic Value of Circulating Cell-Free HPV DNA in Plasma from Cervical Cancer Patients.* Cells, 2022. **11**(14).

^11^ Based on pathological revision after surgery, patient was categorized as having intermediate risk factors and was recommended adjuvant radiochemotherapy with EBRT+Cis but refused to receive this treatment. For the purpose of this study focusing on ccfHPV DNA as a possible biomarker for initial stage, the patient is still categorized as being in the “Primary surgery+adjuvant oncology subgroup”

^12^ Based on pathological revision after surgery, patient was categorized as having intermediate risk factors. See Bhatla, N., et al., *Revised FIGO staging for carcinoma of the cervix uteri.* Int J Gynaecol Obstet, 2019. **145**(1): p. 129-135.

^13^ Based on pathological revision after surgery, patient was categorized as having high-risk factors. See Bhatla, N., et al., *Revised FIGO staging for carcinoma of the cervix uteri.* Int J Gynaecol Obstet, 2019. **145**(1): p. 129-135.

^14^ Not applicable.

| Table S2. Background reads in cases with non-HPV16 related cancer (N=63) | | |
| --- | --- | --- |
| Pt no | HPV tissue^1^ | NGS HPV: HPV16 DNA (reads)^2^ |
| 1 | HPV18 | 0 |
| 2 | HPV18 | 1 |
| 3 | HPV18 | 4 |
| 14 | HPV18 | 2 |
| 15 | HPV18 | 3 |
| 16 | HPV18 | 1 |
| 19 | HPV18+HPV45 | 2 |
| 21 | HPV18 | 2 |
| 27 | HPV18 | 0 |
| 61 | HPV45 | 2 |
| 62 | HPV39 | 1 |
| 63 | HPV68 | 2 |
| 65 | HPV18 | 7 |
| 66 | HPV39 | 1 |
| 67 | HPV31 | 2 |
| 68 | HPV45 | 1 |
| 69 | HPV33 | 1 |
| 71 | HPV45 | 3 |
| 72 | HPV45 | 26 |
| 74 | HPV31 | 1 |
| 77 | HPV18 | 0 |
| 78 | HPV18 | 1 |
| 80 | HPV45 | 3 |
| 40 | HPV18 | 3 |
| 44 | HPV18 | 2 |
| 59 | HPV18 | 2 |
| 83 | HPV18 | 7 |
| 84 | HPV45 | 1 |
| 86 | HPV18 | 3 |
| 87 | HPV45 | 11 |
| 89 | HPV31 | 0 |
| 90 | HPV18 | 2 |
| 91 | HPV18 | 1 |
| 92 | HPV33 | 9 |
| 94 | HPV35 | 5 |
| 98 | HPV73 | 1 |
| 99 | HPV18 | 2 |
| 101 | HPV33 | 7 |
| 102 | HPV30 | 6 |
| 103 | HPV58 | 3 |
| 104 | HPV45 | 4 |
| 105 | HPV33 | 1 |
| 106 | HPV30+HPV33 | 1 |
| 108 | HPV11 | 1 |
| 109 | HPV39 | 2 |
| 110 | HPV33 | 2 |
| 111 | HPV45 | 5 |
| 112 | HPV45 | 4 |
| 114 | HPV33 | 3 |
| 115 | HPV52 | 0 |
| 117 | HPV45 | 1 |
| 121 | HPV45 | 0 |
| 122 | HPV18 | 5 |
| 123 | HPV45 | 3 |
| 124 | HPV31 | 2 |
| 125 | HPV70 | 1 |
| 128 | HPV18 | 0 |
| 131 | HPV31 | 2 |
| 132 | HPV31 | 2 |
| 134 | HPV33 | 3 |
| 135 | HPV45 | 0 |
| 136 | HPV45 | 2 |
| 138 | HPV18 | 0 |
| LoD^3^ = 20.4 | | |

^1^ HPV genotype detected with the NGS HPV genotyping assay described in Andersen K, Holm K, Tranberg M, Pedersen CL, Bonlokke S, Steiniche T, et al. Targeted Next Generation Sequencing for Human Papillomavirus Genotyping in Cervical Liquid-Based Cytology Samples. Cancers (Basel). 2022;14(3).

^2^ Reads describes the highest number of reads detected in all of the HPV16 amplicons detected with the NGS HPV genotyping panel.

^3^ Calculation of LoD for tissue samples were done using the same approach as in Andersen K, Holm K, Tranberg M, Pedersen CL, Bonlokke S, Steiniche T, et al. Targeted Next Generation Sequencing for Human Papillomavirus Genotyping in Cervical Liquid-Based Cytology Samples. Cancers (Basel). 2022;14(3). Thus, since HPV16 was the most prevalent HPV type and the HPV type with the highest number of reads, the LoD was based on this type, creating the most background reads. We therefore used tissue from all CC cases with non-HPV16 related cancer (non-HPV16 cases) to assess the amount of background in the HPV16 amplicons (see ^2^). LoD was calculated as follows: *LoD = mean_non-HPV16 cases_ + (2 * SD_non-HPV16 cases_*), and we subsequently established a conservative value for the LoD by using twice the amount of this.

| Table S3. Qualitative assessment of ccfHPV DNA using NGS HPV genotyping on 25 CIN3 patients and 15 negative controls. | | |
| --- | --- | --- |
| *CIN3 patients* | | |
| Pt no. | HPV tissue^1^ | NGS HPV: ccfHPV DNA (reads)^2^ |
| CIN3 1 | HPV33 | 1 |
| CIN3 2 | HPV16 | 0 |
| CIN3 3 | HPV45 | 0 |
| CIN3 4 | HPV58 | 1 |
| CIN3 5 | HPV16 | 0 |
| CIN3 6 | HPV52 | 0 |
| CIN3 7 | HPV16+HPV52 | 0 |
| CIN3 8 | HPV31 | 1 |
| CIN3 9 | HPV52 | 1 |
| CIN3 10 | HPV16 | 0 |
| CIN 3 11 | HPV16 | 0 |
| CIN3 12 | HPV51 | 3 |
| CIN3 13 | HPV70 | 1 |
| CIN3 14 | HPV58 | 0 |
| CIN3 15 | HPV16 | 0 |
| CIN3 16 | HPV16 | 1 |
| CIN3 17 | HPV31 | 1 |
| CIN3 18 | HPV59 | 2 |
| CIN3 19 | HPV33+HPV39 | 1 |
| CIN3 20 | HPV56+HPV73 | 0 |
| CIN3 21 | HPV35 | 1 |
| CIN3 22 | HPV58 | 2 |
| CIN3 23 | HPV31 | 0 |
| CIN3 24 | HPV16 | 1 |
| CIN3 25 | HPV52 | 2 |
| *Negative controls* | | |
| Pt no. | HPV tissue | NGS HPV: ccfHPV DNA (reads)^2^ |
| C1 | N/A | 1 |
| C2 | N/A | 0 |
| C3 | N/A | 1 |
| C4 | N/A | 1 |
| C5 | N/A | 0 |
| C6 | N/A | 0 |
| C7 | N/A | 0 |
| C8 | N/A | 2 |
| C9 | N/A | 0 |
| C10 | N/A | 1 |
| C11 | N/A | 1 |
| C12 | N/A | 1 |
| C13 | N/A | 1 |
| C14 | N/A | 0 |
| C15 | N/A | 0 |
| LoD^3^ = 4 | | |

^1^ HPV genotype detected with the NGS HPV genotyping assay described in Andersen K, Holm K, Tranberg M, Pedersen CL, Bonlokke S, Steiniche T, et al. Targeted Next Generation Sequencing for Human Papillomavirus Genotyping in Cervical Liquid-Based Cytology Samples. Cancers (Basel). 2022;14(3).

^2^ Reads describes the highest number of reads detected in all of the amplicons detected with the NGS HPV genotyping panel.

^3^ Calculation of LoD for plasma was done by using results from negative controls to assess the amount of background in the amplicons. LoD was calculated as follows: *LoD = mean_neg controls_ + (2 * SD_negative controls_*), and as with LoD for tissue, we subsequently established a conservative value for the LoD by using twice the amount of this.

| Table S4. ccfHPV DNA positive patients from the case cohort with results closest cut-off for HPV positivity (n=14)^1^ | | | |
| --- | --- | --- | --- |
| Pt no | HPV tissue^2^ | NGS HPV: ccfHPV DNA positive number of amplicons^3^ | NGS HPV: ccfHPV DNA reads^4^ |
| 17 | HPV16 | 4 | 17, 30, 50, and 56 HPV16 reads |
| 39 | HPV16 | 3 | 10, 16, and 20 HPV16 reads |
| 40 | HPV18 | 4 | 24, 25, 60 and 64 HPV18 reads |
| 42 | HPV16 | 2 | 26 and 26 HPV16 reads |
| 43 | HPV16 | 3 | 14, 20, and 64 HPV16 reads |
| 49 | HPV16 | 5 | 11, 26, 35, 38, and 47 HPV16 reads |
| 58 | HPV16 | 3 | 5, 5, and 7 HPV16 reads |
| 84 | HPV45 | 2 | 19 and 64 HPV45 reads |
| 93 | HPV16 | 4 | 24, 29, 30, and 38 HPV16 reads |
| 113 | HPV33 | 2 | 15 and 16 HPV33 reads |
| 114 | HPV33 | 4 | 3, 11, 12, and 17 HPV33 reads |
| 115 | HPV52 | 4 | 4, 7, 11, and 12 HPV52 reads |
| 128 | HPV18 | 3 | 19, 27, and 53 HPV18 reads |
| 129 | HPV16 | 3 | 11, 21, and 32 HPV16 reads |

^1^ Data from the NGS plasma analyses with results closest to the calculated cut-off for HPV positivity (i.e. ≥2 amplicons with >2 reads).

^2^ HPV genotype detected with the NGS HPV genotyping assay described in Andersen K, Holm K, Tranberg M, Pedersen CL, Bonlokke S, Steiniche T, et al. Targeted Next Generation Sequencing for Human Papillomavirus Genotyping in Cervical Liquid-Based Cytology Samples. Cancers (Basel). 2022;14(3).

^3^ Number of ccfHPV DNA positive amplicons with the NGS Genotyping panel according to cut-off for HPV DNA positivity (i.e. ≥2 amplicons with >2 reads).

^4^ Number of reads detected in the HPV positive amplicons.

| Table S5. ccfHPV^neg^ patients from the case cohort with any detectable HPV reads (n=16)^1^ | | | |
| --- | --- | --- | --- |
| Pt no | HPV tissue^2^ | Number of amplicons with detectable reads^3^ | NGS HPV: ccfHPV DNA reads^4^ |
| 6 | HPV16 | 1 | 1 HPV16 read |
| 8 | HPV16 | 1 | 19 HPV16 reads |
| 11 | HPV16 | 2 | 1 and 2 HPV16 reads |
| 29 | HPV16 | 1 | 29 HPV16 reads |
| 30 | HPV16 | 1 | 1 HPV16 read |
| 46 | HPV16 | 1 | 16 HPV16 reads |
| 50 | HPV16 | 1 | 1 HPV16 read |
| 75 | HPV16 | 1 | 1 HPV16 reads |
| 76 | HPV16+HPV67 | 2 | 1 and 1 HPV16 read |
| 78 | HPV18 | 1 | 1 HPV18 read |
| 79 | HPV16 | 2 | 1 and 1 HPV16 read |
| 82 | HPV16 | 2 | 1 and 2 HPV16 reads |
| 90 | HPV18 | 1 | 15 HPV18 reads |
| 102 | HPV30 | 1 | 31 HPV30 reads |
| 118 | HPV16 | 2 | 1 and 19 HPV16 reads |
| 135 | HPV45 | 1 | 15 HPV45 reads |

^1^ Data from the NGS plasma analyses of ccfHPV^neg^ patients with any detectable reads in the eight amplicons detected for each HPV genotype with the NGS HPV genotyping panel.

^2^ HPV genotype detected with the NGS HPV genotyping assay described in Andersen K, Holm K, Tranberg M, Pedersen CL, Bonlokke S, Steiniche T, et al. Targeted Next Generation Sequencing for Human Papillomavirus Genotyping in Cervical Liquid-Based Cytology Samples. Cancers (Basel). 2022;14(3).

^3^ Number of amplicons with any detectable reads in the eight amplicons detected for each HPV genotype with the NGS HPV genotyping panel.

^4^ Number of reads detected in the HPV amplicons.

| Table S6. Qualitative assessment of tissue HPV DNA using NGS HPV16 panel on ten cases with non-HPV16 related cervical cancer (n=10)^1^ | | |
| --- | --- | --- |
| Pt no | HPV tissue^2^ | NGS HPV16 panel (reads)^3^ |
| 40 | HPV18 | 6 |
| 98 | HPV73 | 6 |
| 102 | HPV30 | 0 |
| 105 | HPV33 | 0 |
| 108 | HPV11 | 1 |
| 109 | HPV39 | 0 |
| 111 | HPV45 | 0 |
| 115 | HPV52 | 0 |
| 124 | HPV31 | 7 |
| 125 | HPV70 | 2 |
| LoD^4^ = 15.6 | | |

^1^ Data from 10 selected cases with non-HPV16 related cervical cancer. Cases were chosen based on detection of different HPV genotypes in tissue analyses with the NGS Genotyping panel.

^2^ HPV genotype detected with the NGS HPV genotyping assay described in Andersen K, Holm K, Tranberg M, Pedersen CL, Bonlokke S, Steiniche T, et al. Targeted Next Generation Sequencing for Human Papillomavirus Genotyping in Cervical Liquid-Based Cytology Samples. Cancers (Basel). 2022;14(3).

^3^ Reads describes the highest number of reads detected in all of the amplicons detected with the NGS HPV16 panel.

^4^*,* Calculation of LoD for tissue was done by using tissue from patients with non-HPV16 related CC. By means of these, the amount of background reads was assessed. NGS results from ten non-HPV16 cases were used, and these were chosen based on detection of different HPV genotypes in tissue analyses with the NGS HPV genotyping panel. LoD for all 104 amplicons was calculated as follows: *LoD = mean_non-HPV16 cases_ + (2 * SD_non-HPV16 cases_),* and as with LoDs with the NGS HPV Genotyping panel, we subsequently established a conservative value for the LoD by using twice the amount of this.

| Table S7. Qualitative assessment of ccfHPV DNA^1^ | | | | | | | |
| --- | --- | --- | --- | --- | --- | --- | --- |
| *Primary surgery subgroup (n=50)* | | | | | | | |
| Pt no | HPV tissue^2^ | ddPCR: Input (ng) | ddPCR: ccfHPV DNA (copies/ml)^3^ | ddPCR: ccHPV DNA pos/neg as by cutoff^4^ | NGS: Input (ng) | NGS HPV: ccfHPV DNA (reads)^5^ | NGS HPV: ccfHPV DNA pos/neg as by cutoff^6^ |
| 1 | HPV18 | 20.3 | 0 | Neg | 40.7 | 0 | Neg |
| 2 | HPV18 | 31.2 | 0 | Neg | 60 | 0 | Neg |
| 3 | HPV18 | 3.5 | 0 | Neg | 7.0 | 0 | Neg |
| 4 | HPV16 | 3.9 | 1 | Neg | 7.8 | 0 | Neg |
| 5 | HPV16 | 6.3 | 0 | Neg | 12.5 | 0 | Neg |
| 6 | HPV16 | 3.4 | 0 | Neg | 6.8 | 0 | Neg |
| 7 | HPV16 | 2.5 | 0 | Neg | 5.0 | 0 | Neg |
| 8 | HPV16 | 2.5 | 0 | Neg | 5.0 | 0 | Neg |
| 9 | HPV16 | 3.2 | 0 | Neg | 6.4 | 0 | Neg |
| 10 | HPV16 | 2.8 | 1 | Neg | 5.6 | 0 | Neg |
| 12 | HPV16 | 5.0 | 0 | Neg | 10.0 | 0 | Neg |
| 13 | HPV16 | 9.6 | 2 | Neg | 19.3 | 0 | Neg |
| 14 | HPV18 | 2.5 | 0 | Neg | 5.0 | 0 | Neg |
| 15 | HPV18 | 2.3 | 0 | Neg | 4.6 | 0 | Neg |
| 16 | HPV18 | 4.1 | 0 | Neg | 8.2 | 0 | Neg |
| 17 | HPV16 | 1.8 | 3 | Neg | 3.5 | 0-56 | Pos HPV16 |
| 18 | HPV16 | 97.2 | 0 | Neg | 7.4 | 0 | Neg |
| 19 | HPV18+HPV45 | 117.0 | 0 | Neg | 9.7 | 0 | Neg |
| 20 | HPV16+HPV33 | 102.3 | 0 | Neg | 7.3 | 0 | Neg |
| 21 | HPV18 | 126.3 | 0 | Neg | 53.6 | 0 | Neg |
| 22 | HPV16 | 97.2 | 1 | Neg | 48.5 | 0-115 | Pos HPV16 |
| 23 | HPV16 | 91.2 | 1 | Neg | 9.1 | 0 | Neg |
| 24 | HPV16 | 107.1 | 1 | Neg | 5.4 | 0 | Neg |
| 25 | HPV16 | 114.6 | 1 | Neg | 5.5 | 0 | Neg |
| 26 | HPV16 | 88.8 | 13 | Pos | 5.4 | 0 | Neg |
| 27 | HPV18 | 78.6 | 0 | Neg | 7.5 | 0 | Neg |
| 28 | HPV16 | 81.3 | 2 | Neg | 4.1 | 0 | Neg |
| 29 | HPV16 | 62.1 | 5 | Pos | 3.9 | 0 | Neg |
| 30 | HPV16 | 83.4 | 4 | Pos | 9.1 | 0-1 | Neg |
| 61 | HPV45 |  | - | - | 5.2 | 0 | Neg |
| 62 | HPV39 |  | - | - | 5.2 | 0 | Neg |
| 63 | HPV68 |  | - | - | 13.7 | 0 | Neg |
| 64 | HPV16 |  | - | - | 7.5 | 0 | Neg |
| 65 | HPV18 |  | - | - | 8.3 | 0 | Neg |
| 66 | HPV39 |  | - | - | 7.9 | 0 | Neg |
| 67 | HPV31 |  | - | - | 9.2 | 0 | Neg |
| 68 | HPV45 |  | - | - | 4.8 | 0 | Neg |
| 69 | HPV33 |  | - | - | 20.8 | 0 | Neg |
| 70 | HPV16+HPV33 |  | - | - | 5.9 | 0 | Neg |
| 71 | HPV45 |  | - | - | 7.0 | 0-38 | Pos HPV45 |
| 72 | HPV45 |  | - | - | 60.0 | 0 | Neg |
| 73 | HPV16 |  | - | - | 10.2 | 0-81 | Pos HPV16 |
| 74 | HPV31 |  | - | - | 9.2 | 0 | Neg |
| 75 | HPV16 |  | - | - | 16.6 | 0-1 | Neg |
| 76 | HPV16+HPV67 |  | - | - | 4.4 | 0-1 | Neg |
| 77 | HPV18 |  | - | - | 10.8 | 0 | Neg |
| 78 | HPV18 |  | - | - | 5.8 | 0-1 | Neg |
| 79 | HPV16 |  | - | - | 4.9 | 0-1 | Neg |
| 80 | HPV45 |  | - | - | 4.5 | 0 | Neg |
| 81 | HPV45+HPV16 |  | - | - | 6.1 | 0 | Neg |
| *Primary surgery+adjuvant oncology subgroup (n=22)* | | | | | | | |
| Pt no | HPV tissue | ddPCR: Input (ng) | ddPCR: ccfHPV DNA (copies/ml) | ddPCR: ccHPV DNA pos/neg as by cutoff | NGS: Input (ng) | NGS HPV: ccfHPV DNA (reads) | NGS HPV: ccfHPV DNA pos/neg as by cutoff |
| 11 | HPV16 | 3.0 | 0 | Neg | 6.0 | 0-2 | Neg |
| 82 | HPV16 |  | - | - | 60 | 0-2 | Neg |
| 83 | HPV18 |  | - | - | 25.9 | 0 | Neg |
| 84 | HPV45 |  | - | - | 16.4 | 0-64 | Pos HPV45 |
| 85 | HPV16 |  | - | - | 8.6 | 213-3141 | Pos HPV16 |
| 86 | HPV18 |  | - | - | 5.4 | 0 | Neg |
| 87 | HPV45 |  | - | - | 5.6 | 0 | Neg |
| 88 | HPV16+HPV70 |  | - | - | 5.4 | 12-145 | Pos HPV16 |
| 89 | HPV31 |  | - | - | 11.3 | 0 | Neg |
| 90 | HPV18 |  | - | - | 9.5 | 0-15 | Neg |
| 91 | HPV18 |  | - | - | 8.4 | 0 | Neg |
| 92 | HPV33 |  | - | - | 10.3 | 0 | Neg |
| 93 | HPV16 |  | - | - | 7.5 | 0-38 | Pos HPV16 |
| 94 | HPV35 |  | - | - | 8.9 | 43-674 | Pos HPV35 |
| 95 | HPV16 |  | - | - | 9.8 | 0 | Neg |
| 96 | HPV16+HPV18 |  | - | - | 4.5 | 0 | Neg |
| 97 | HPV16 |  | - | - | 5.2 | 18-170 | Pos HPV16 |
| 98 | HPV73 |  | - | - | 4.4 | 0 | Neg |
| 99 | HPV18 |  | - | - | 9.5 | 0 | Neg |
| 100 | HPV16 |  | - | - | 6.6 | 201-683 | Pos HPV16 |
| 101 | HPV33 |  | - | - | 11.8 | 4-131 | Pos HPV33 |
| 102 | HPV30 |  | - | - | 6.1 | 0-31 | Neg |
| *Primary oncology subgroup (n=67)* | | | | | | | |
| Pt no | HPV tissue | ddPCR: Input (ng) | ddPCR: ccfHPV DNA (copies/ml) | ddPCR: ccHPV DNA pos/neg as by cutoff | NGS: Input (ng) | NGS HPV: ccfHPV DNA (reads) | NGS HPV: ccfHPV DNA pos/neg as by cutoff |
| 31 | HPV16 | 33.6 | 24 | Pos | 60 | 11-145 | Pos HPV16 |
| 32 | HPV16 | 114.6 | 541 | Pos | 60 | 1572-3990 | Pos HPV16 |
| 33 | HPV16 | 3.6 | 88 | Pos | 7.3 | 2213-6572 | Pos HPV16 |
| 34 | HPV16 | 5.6 | 41 | Pos | 11.3 | 752-2396 | Pos HPV16 |
| 35 | HPV16 | 1.8 | 0 | Neg | 4.0 | 0 | Neg |
| 36 | HPV16 | 2.7 | 2 | Neg | 5.4 | 26-321 | Pos HPV16 |
| 37 | HPV16 | 3.5 | 186 | Pos | 6.9 | 1331-4854 | Pos HPV16 |
| 38 | HPV16 | 4.5 | 54 | Pos | 9.0 | 286-3834 | Pos HPV16 |
| 39 | HPV16 | 2.7 | 0 | Neg | 5.3 | 0-20 | Pos HPV16 |
| 40 | HPV18 | 3.4 | 1.5 | Neg | 6.8 | 24-64 | Pos HPV18 |
| 41 | HPV16 | 4.4 | 76 | Pos | 4.5 | 599-2041 | Pos HPV16 |
| 42 | HPV16 | 7.1 | 0 | Neg | 14.2 | 0-26 | Pos HPV16 |
| 43 | HPV16 | 6.5 | 1 | Neg | 12.9 | 0-64 | Pos HPV16 |
| 44 | HPV18 | 5.6 | 0 | Neg | 7.1 | 0 | Neg |
| 45 | HPV16 | 3.6 | 4 | Pos | 7.1 | 32-91 | Pos HPV16 |
| 46 | HPV16 | 9.3 | 0 | Neg | 18.6 | 0-16 | Neg |
| 47 | HPV16 | 5.2 | 28 | Pos | 10.4 | 0-1198 | Pos HPV16 |
| 48 | HPV16 | 7.9 | 58 | Pos | 15.8 | 423-1845 | Pos HPV16 |
| 49 | HPV16 | 8.7 | 1 | Neg | 17.4 | 0-47 | Pos HPV16 |
| 50 | HPV16 | 4.9 | 0 | Neg | 9.8 | 0-1 | Neg |
| 51 | HPV16 | 4.1 | 43 | Pos | 8.2 | 972-3578 | Pos HPV16 |
| 52 | HPV16 | 4.4 | 30 | Pos | 8.7 | 213-1367 | Pos HPV16 |
| 53 | HPV16 | 78.6 | 4 | Pos | 5.3 | 0-137 | Pos HPV16 |
| 54 | HPV16 | 5.4 | 181 | Pos | 10.9 | 1046-6865 | Pos HPV16 |
| 55 | HPV16 | 82.2 | 9 | Pos | 3.0 | 76-587 | Pos HPV16 |
| 56 | HPV16 | 90.3 | 11 | Pos | 7.3 | 0-609 | Pos HPV16 |
| 57 | HPV16 | 105.6 | 208 | Pos | 7.6 | 5000-18200 | Pos HPV16 |
| 58 | HPV16 | 86.1 | 136 | Pos | 12.2 | 0-7 | Pos HPV16 |
| 59 | HPV18 | 84.9 | 0 | Neg | 4.8 | 37-404 | Pos HPV18 |
| 60 | HPV16 | 84.0 | 810 | Pos | 5.4 | 7738-48512 | Pos HPV16 |
| 103 | HPV58 |  | - | - | 14.4 | 49-19564 | Pos HPV58 |
| 104 | HPV45 |  | - | - | 6.8 | 4-263 | Pos HPV45 |
| 105 | HPV33 |  | - | - | 5.7 | 241-575 | Pos HPV33 |
| 106 | HPV30+HPV33 |  | - | - | 11.0 | 2-157 | Pos HPV33 |
| 107 | HPV16 |  | - | - | 60.0 | 0 | Neg |
| 108 | HPV11 |  | - | - | 21.2 | 4-175 | Pos HPV11 |
| 109 | HPV39 |  | - | - | 11.3 | 7-155 | Pos HPV39 |
| 110 | HPV33 |  | - | - | 6.7 | 1235-3923 | Pos HPV33 |
| 111 | HPV45 |  | - | - | 7.3 | 0 | Neg |
| 112 | HPV45 |  | - | - | 10.8 | 0-120 | Pos HPV45 |
| 113 | HPV45+HPV16 |  | - | - | 11.3 | 0 | Neg |
| 114 | HPV33 |  | - | - | 7.4 | 0-1431 | Pos HPV33 |
| 115 | HPV52 |  | - | - | 8.0 | 0-12 | Pos HPV52 |
| 116 | HPV16 |  | - | - | 12.5 | 1129-4119 | Pos HPV16 |
| 117 | HPV45 |  | - | - | 4.9 | 0-6948 | Pos HPV45 |
| 118 | HPV16 |  | - | - | 13.5 | 0-19 | Neg |
| 119 | HPV16 |  | - | - | 12.3 | 577-2434 | Pos HPV16 |
| 120 | HPV16 |  | - | - | 6.6 | 196-520 | Pos HPV16 |
| 121 | HPV45 |  | - | - | 2.6 | 0 | Neg |
| 122 | HPV18 |  | - | - | 8.3 | 0-191 | Pos HPV18 |
| 123 | HPV45 |  | - | - | 15.7 | 0-468 | Pos HPV45 |
| 124 | HPV45 |  | - | - | 7.1 | 1222-6469 | Pos HPV31 |
| 125 | HPV31 |  | - | - | 8.1 | 34-9223 | Pos HPV70 |
| 126 | HPV16 |  | - | - | 9.7 | 942-5810 | Pos HPV16 |
| 127 | HPV16 |  | - | - | 11.8 | 7605-18974 | Pos HPV16 |
| 128 | HPV18 |  | - | - | 6.7 | 0-53 | Pos HPV18 |
| 129 | HPV16 |  | - | - | 11.7 | 0-32 | Pos HPV16 |
| 130 | HPV16 |  | - | - | 4.4 | 3167-12801 | Pos HPV16 |
| 131 | HPV31 |  | - | - | 6.3 | 2146-4583 | Pos HPV31 |
| 132 | HPV31 |  | - | - | 12.7 | 0 | Neg |
| 133 | HPV18+HPV16 |  | - | - | 9.3 | 0-323 | Pos HPV18 |
| 134 | HPV33 |  | - | - | 56.6 | 0 | Neg |
| 135 | HPV45 |  | - | - | 11.9 | 0-15 | Neg |
| 136 | HPV45 |  | - | - | 7.6 | 0 | Neg |
| 137 | HPV45+HPV16 |  | - | - | 8.2 | 0-192 | Pos HPV45 |
| 138 | HPV18 |  | - | - | 5.6 | 0-243 | Pos HPV18 |
| 139 | HPV16 |  | - | - | 11.5 | 1-159 | Pos HPV16 |

^1^ For patient no. 1-60, ccfHPV DNA was also measured by means of ddPCR in Bonlokke, S., et al., *The Diagnostic Value of Circulating Cell-Free HPV DNA in Plasma from Cervical Cancer Patients.* Cells, 2022. **11**(14).

^2^ HPV genotype detected with the NGS HPV genotyping assay described in Andersen K, Holm K, Tranberg M, Pedersen CL, Bonlokke S, Steiniche T, et al. Targeted Next Generation Sequencing for Human Papillomavirus Genotyping in Cervical Liquid-Based Cytology Samples. Cancers (Basel). 2022;14(3).

^3^ Data on ddPCR results are extracted from Table S1 in Bonlokke, S., et al., *The Diagnostic Value of Circulating Cell-Free HPV DNA in Plasma from Cervical Cancer Patients.* Cells, 2022. **11**(14).

^4^ Cutoff for ccfHPV DNA positivity is > 3 copies/ml plasma.

^5^ Reads describes the range for detected reads in the eight amplicons detected for each HPV genotype with the NGS HPV genotyping panel.

^6^ Based on analyses of cell-free DNA from negative controls (see Table S2), cutoff for HPV DNA positivity with the NGS HPV genotyping panel is defined as ≥2 amplicons for a given HPV genotype with >4 reads detected (see manuscript and Table S3).

| Table S8: Analyses of tissue samples from the 67 cases positive for HPV16 (single genotype infection) in tumor tissue using NGS HPV16 panel | | | |
| --- | --- | --- | --- |
| *Primary surgery patients (N = 23)* | | | |
| Pt no. | Tissue: NGS HPV genotyping panel^1^ | Tissue: NGS HPV16 pos/neg as by cutoff^2^ | Tissue: HPV16 integration status^3^ |
| 4 | HPV16 | HPV16 | Non-integration |
| 5 | HPV16 | HPV16 | Non-integration |
| 6 | HPV16 | HPV16 | Complete integration (loss of E1_24-L2_19) |
| 7 | HPV16 | HPV16 | Complete integration (loss of E2_9-L2_8) |
| 8 | HPV16 | HPV16 | Non-integration |
| 9 | HPV16 | HPV16 | Non-integration |
| 10 | HPV16 | HPV16 | Non-integration |
| 12 | HPV16 | HPV16 | Non-integration |
| 13 | HPV16 | HPV16 | Non-integration |
| 17 | HPV16 | HPV16 | Non-integration |
| 18 | HPV16 | HPV16 | Non-integration |
| 22 | HPV16 | HPV16 | Non-integration |
| 23 | HPV16 | HPV16 | Non-integration |
| 24 | HPV16 | HPV16 | Non-integration |
| 25 | HPV16 | HPV16 | Non-integration |
| 26 | HPV16 | HPV16 | Non-integration |
| 28 | HPV16 | HPV16 | Complete integration (loss of E1_3-E1_5) |
| 29 | HPV16 | HPV16 | Non-integration |
| 30 | HPV16 | HPV16 | Complete integration (loss of E2_5-E2_7) |
| 64 | HPV16 | HPV16 | Non-integration |
| 73 | HPV16 | HPV16 | Non-integration |
| 75 | HPV16 | HPV16 | Non-integration |
| 79 | HPV16 | HPV16 | Non-integration |
| *Primary surgery+adjuvant oncology patients (N = 7)* | | | |
| 11 | HPV16 | HPV16 | Complete integration (loss of E1_24-L2_3) |
| 82 | HPV16 | HPV16 | Non-integration |
| 85 | HPV16 | HPV16 | Non-integration |
| 93 | HPV16 | HPV16 | Complete integration (loss of E1_22-L2_5) |
| 95 | HPV16 | HPV16 | Non-integration |
| 97 | HPV16 | HPV16 | Non-integration |
| 100 | HPV16 | HPV16 | Non-integration |
| *Primary oncology patients (N = 37)* | | | |
| 31 | HPV16 | HPV16 | Non-integration |
| 32 | HPV16 | HPV16 | Non-integration |
| 33 | HPV16 | HPV16 | Complete integration (loss of E1_19-22 and L2_13) |
| 34 | HPV16 | HPV16 | Non-integration |
| 35 | HPV16 | HPV16 | Complete integration (loss of E2_7-E2.E5) |
| 36 | HPV16 | HPV16 | Non-integration |
| 37 | HPV16 | HPV16 | Non-integration |
| 38 | HPV16 | HPV16 | Non-integration |
| 39 | HPV16 | HPV16 | Complete integration (loss of E1_17-E1_25) |
| 41 | HPV16 | HPV16 | Non-integration |
| 42 | HPV16 | HPV16 | Non-integration |
| 43 | HPV16 | HPV16 | Non-integration |
| 45 | HPV16 | HPV16 | Non-integration |
| 46 | HPV16 | HPV16 | Complete integration (loss of E1E2_2-E2_5) |
| 47 | HPV16 | HPV16 | Non-integration |
| 48 | HPV16 | HPV16 | Complete integration (loss of E1E2_2-E2_5) |
| 49 | HPV16 | HPV16 | Complete integration (loss of E1_19-L2L1_2) |
| 50 | HPV16 | HPV16 | Complete integration (loss of L2_17) |
| 51 | HPV16 | HPV16 | Non-integration |
| 52 | HPV16 | HPV16 | Complete integration (loss of L2_17) |
| 53 | HPV16 | HPV16 | Non-integration |
| 54 | HPV16 | HPV16 | Non-integration |
| 55 | HPV16 | HPV16 | Non-integration |
| 56 | HPV16 | HPV16 | Non-integration |
| 57 | HPV16 | HPV16 | Non-integration |
| 58 | HPV16 | HPV16 | Complete integration (loss of E1_18-L2_8) |
| 60 | HPV16 | HPV16 | Non-integration |
| 107 | HPV16 | HPV16 | Non-integration |
| 116 | HPV16 | HPV16 | Non-integration |
| 118 | HPV16 | HPV16 | Complete integration (loss of E1_3-E5_1) |
| 119 | HPV16 | HPV16 | Non-integration |
| 120 | HPV16 | HPV16 | Complete integration (loss of L2_1-L2_7) |
| 126 | HPV16 | HPV16 | Non-integration |
| 127 | HPV16 | HPV16 | Non-integration |
| 129 | HPV16 | HPV16 | Complete integration (loss of E1..E2_2-L1_3) |
| 130 | HPV16 | HPV16 | Non-integration |
| 139 | HPV16 | HPV16 | Complete integration (loss of E2_5-E2_4) |

^1^ HPV genotype detected with the NGS HPV genotyping assay described in Andersen K, Holm K, Tranberg M, Pedersen CL, Bonlokke S, Steiniche T, et al. Targeted Next Generation Sequencing for Human Papillomavirus Genotyping in Cervical Liquid-Based Cytology Samples. Cancers (Basel). 2022;14(3).

^2^ HPV genotype results with the NGS HPV16 panel

^3^ HPV16 integration status defined as follows; complete integration: ≥2 consecutive amplicons with ≤15 reads; non-integration: <2 consecutive amplicons with >15 reads.

| Table S9. Disease burden in primary oncology patients according to HPV16 integration status | | | |
| --- | --- | --- | --- |
|  | Primary oncology patients with complete HPV16 integration  (*N* = 13) | Primary oncology patients with HPV16 non-integration  (*N* = 24) | P-value |
| Tumor size (mm)  Mean ± SD | 48.8 ± 12.1 | 54.33 ± 19.2 | *p* = 0.31^1^ |
| *FIGO 2018* | n (%) | n (%) | *p* = 0.97^2^ |
| IB3 | 1 (7.7) | 1 (4.2) |  |
| IIB | 5 (38.5) | 10 (41.7) |  |
| IIIC1 | 6 (46.2) | 10 (41.7) |  |
| IIIC2 | 0 (0.0) | 2 (83.3) |  |
| IVA | 1 (7.7) | 1 (4.2) |  |

^1^ Un-paired two-sided T-test.

^2^ Fisher’s exact test.
